# Gating choreography and mechanism of the human proton-activated chloride channel ASOR

**DOI:** 10.1126/sciadv.abm3942

**Published:** 2022-02-02

**Authors:** Chongyuan Wang, Maya M. Polovitskaya, Bryce D. Delgado, Thomas J. Jentsch, Stephen B. Long

**Affiliations:** 1 Structural Biology Program, Memorial Sloan Kettering Cancer Center, 1275 York Avenue, New York, NY 10065, USA.; 2 Leibniz-Forschungsinstitut für Molekulare Pharmakologie (FMP) and Max-Delbrück-Centrum für Molekulare Medizin (MDC), D-13125 Berlin, Germany.; 3 Graduate Program in Biochemistry and Structural Biology, Cell and Developmental Biology, and Molecular Biology, Weill Cornell Medicine Graduate School of Medical Sciences, New York, NY 10065, USA.; 4 NeuroCure Cluster of Excellence, Charité Universitätsmedizin, D-10117 Berlin, Germany.

## Abstract

The proton-activated chloride channel ASOR (TMEM206/PAC) permeates anions across cellular membranes in response to acidification, thereby enhancing acid-induced cell death and regulating endocytosis. The molecular mechanisms of pH-dependent control are not understood, in part because structural information for an activated conformation of ASOR is lacking. Here, we reconstitute function from purified protein and present a 3.1-Å-resolution cryo–electron microscopy structure of human ASOR at acidic pH in an activated conformation. The work contextualizes a previous acidic pH structure as a desensitized conformation. Combined with electrophysiological studies and high-resolution structures of resting and desensitized states, the work reveals mechanisms of proton sensing and ion pore gating. Clusters of extracellular acidic residues function as pH sensors and coalesce when protonated. Ensuing conformational changes induce metamorphosis of transmembrane helices to fashion an ion conduction pathway unique to the activated conformation. The studies identify a new paradigm of channel gating in this ubiquitous ion channel.

## Teaser

Proton-dependent changes induce transmembrane metamorphosis to form an ion pore in the ASOR proton-activated chloride channel.

## INTRODUCTION

Chloride is the most abundant anion in animal physiology. Its flow across biological membranes regulates the volume and pH of cells and organelles, electrical excitability and signaling in neurons and muscle, transepithelial transport of salt and water, and blood pressure (*1*–*3*). Most, if not all, mammalian cells express a proton-activated chloride channel in their plasma membrane (*4*–*7*) and in endosomes (*8*). This channel, named the acid-sensitive outwardly rectifying (ASOR) channel (*9*) or proton-activated chloride (PAC) channel (*10*), was recently found to be assembled from TMEM206 proteins (*10*, *11*). The channel has been shown to enhance acid-induced cell death (*9*–*13*) and to regulate endosomal pH and endocytosis (*8*). Furthermore, *Tmem206* disruption in mice protects neurons against ischemic brain injury (*10*). Electrophysiological studies indicate that the ion pore is closed at neutral resting pH and begins to activate at pH ~6.0, with half-maximal activation (pH_50_) of ~5.3 (*5*, *7*, *10*, *11*), but the mechanisms for pH sensing and for the gating of the ion pore are not defined.

Recent cryo–electron microscopy (cryo-EM) structures of the human ASOR channel and a pufferfish ortholog, both at pH 8.0 and determined at 3.5 to 3.6 Å resolution, presumably represent a resting conformation of the channel, in accord with the lack of an ion permeation path through the membrane in the structures (*14*, *15*). A 3.7-Å-resolution cryo-EM structure of the channel at pH 4.0 displays pronounced conformational differences but is difficult to put into functional context because no ion permeation pathway through the membrane exists despite the activating pH (*14*). Because channel activity has not yet been recapitulated from purified protein, it is possible that unidentified subunits may be necessary for channel function. Despite the channel’s emerging physiological importance, without a structure of the channel in a conductive conformation, its mechanisms of gating and activation by extracellular protons remain uncertain.

Unexpectedly, the structures of ASOR revealed a degree of similarity to the acid-sensing ion channel (ASIC) family of cation channels, despite a lack of amino acid sequence conservation (*14*–*16*). Structural comparisons show that both ASOR and ASIC channels assemble as trimers of subunits, both channels contain two transmembrane (TM) helices per subunit, and both have a large extracellular domain (ECD) that contains a prominent core region composed primarily of β sheets. Like ASOR channels, ASIC channels are activated by extracellular acidic pH (*17*). Structural and functional studies of ASIC channels have identified a cluster of acidic amino acids within the upper part of the ECD that operate as proton sensors (*16*, *18*–*20*). Coalescence of these amino acids at low pH induces conformational changes in the ECD that propagate to the TM domain (TMD) and cause the pore to dilate, thereby “opening” the channel and allowing ion permeation (*19*). The cluster of acidic residues in the ECD of ASIC is not present in ASOR, and the domain appendage on which the cluster resides is also absent in ASOR. ASOR must have an alternative mechanism of proton dependence. In addition, different architectures of the TMDs for ASIC and ASOR suggest that the mechanisms of ion pore gating are distinct.

From the structure of ASOR at pH 4.0, a histidine residue located adjacent to the TMD (His^98^) was proposed to function as the primary proton sensor (*14*). However, the absence of an ion conduction path in this structure makes it difficult to discern an activation mechanism from the existing data. The pH response of the channel, with a maximal activation at pH 4.5 (*5*, *10*, *11*), also seems incongruous with a histidine, with a typical p*K*_a_ (where *K*_a_ is the acid dissociation constant) value of 6.0, having such a role, suggesting that other region(s) may function as proton sensor(s).

To further investigate the proton-sensing and gating mechanisms of ASOR, we reconstituted channel function using purified human protein and determined a 3.1-Å-resolution cryo-EM structure of the channel at pH 4.5. Structural and functional characterizations show that this structure, which contains an open ion conduction pore, represents an activated state of the channel. Our data suggest that the previous pH 4.0 structure represents a desensitized conformation. With additional electrophysiological studies and high-resolution structures of the resting and desensitized states, we identify the mechanism of proton sensing, refuting the supposition that His^98^ is the primary proton sensor (*14*) and showing that clusters of acidic residues fulfill this role. The work reveals a mechanism of pore gating that is distinct from other structurally characterized ion channels wherein pronounced metamorphosis of the TMD fashions a pathway for ion conduction when the channel is activated.

## RESULTS AND DISCUSSION

### Functional reconstitution in liposomes

**Fig. 1.**
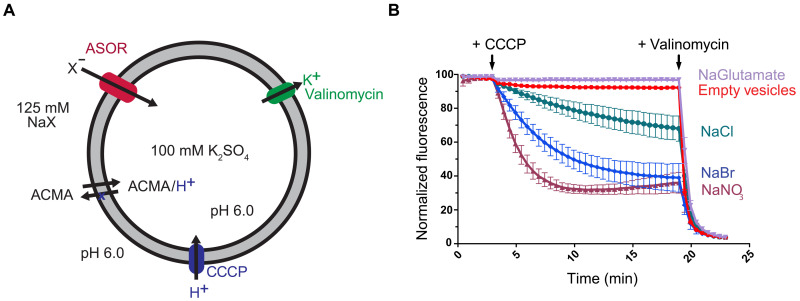
Anion flux through purified ASOR. (**A**) Schematic of the fluorescence-based flux assay. Purified ASOR (TMEM206) was reconstituted into liposomes. Liposomes, which were filled with potassium sulfate, were then diluted into various sodium salts (NaX) to establish ion gradients. Na^+^, K^+^, and sulfate are not readily permeable through the ASOR channel (*5*). Anion influx through the channel (into the liposome) produces a negative electric potential within the liposomes that drives the uptake of protons through an ionophore (CCCP) and quenches the fluorescence of a pH indicator (ACMA). (**B**) Flux measurements. Time-dependent decreases in fluorescence are indicative of anion flux. Glutamate was not detectably permeant. “Empty vesicles” indicate liposomes without protein. Arrows indicate additions of CCCP and the K^+^ ionophore valinomycin. Valinomycin, which causes efflux of K^+^ from the vesicles, was used to establish a fluorescence baseline and to confirm the integrity of liposomes. Fluorescence values were normalized by dividing by the value before CCCP addition and were within ±10% among the experiments. Because ACMA is sensitive to pH, we were not able to use this assay to study the pH-dependent activation of the reconstituted channel.

Full-length human ASOR (TMEM206) protein was expressed in mammalian cells and purified for functional and structural analyses (fig. S1). Reconstitution into liposomes demonstrated that the channel forms a pore permeable to Cl^−^ (Fig. 1). As observed for the native channel or heterologously expressed TMEM206 (*5*, *10*, *11*), the reconstituted channel was also permeable to NO_3_^−^ and Br^−^, with a flux sequence of NO_3_^−^ > Br^−^ > Cl^−^ (Fig. 1). Hence, the purified protein forms a conduction path for anions with hallmarks of the endogenous channel, demonstrating that anion conduction through the channel is intrinsically conferred by the ASOR (TMEM206) polypeptide and does not require additional subunits.

### Resting, activated, and desensitized states

**Fig. 2.**
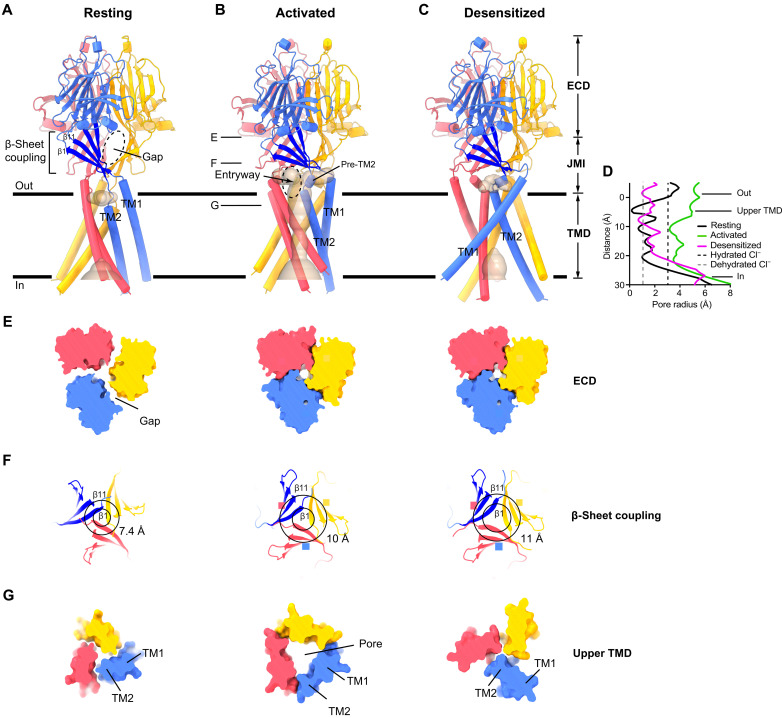
Resting, activated, and desensitized conformations of ASOR. (**A** to **C**) Cartoon representations viewed from the membrane, with subunits colored separately. Solvent-accessible surfaces within the TMD are depicted (wheat color). Only the activated conformation has a continuous pore through the membrane. Horizontal lines indicate approximate boundaries of the membrane. (**D**) Dimensions within the TMD of each structure, calculated along the symmetry axis as the distance to the nearest van der Waals protein contact. Dashed lines indicate the radii of hydrated and dehydrated Cl^−^ ions. (**E** to **G**) Cross sections of the channel, showing dimensional changes in the ECD, β-sheet coupling domain, and upper TMD among the structures (left, resting; middle, activated; right, desensitized). In (E) and (G), 5-Å-thick slices of the molecular surfaces, taken at positions corresponding to “E” and “G” labels from (B) in each of the structures, are shown from the extracellular side. In (F), 10-Å-thick slices of cartoon representations of the β-sheet coupling domains, taken at position corresponding to the “F” label in (B), are shown from an intracellular perspective. Residues marking the ends of the β1 and β11 strands (attaching to TM1 and TM2) are connected by circles. Diameters of the outer circles (for β11) are indicated to highlight dilation of the β-sheet coupling domains in the activated and desensitized structures.

Having identified that purified ASOR protein can form an ion conduction pore, we performed cryo-EM studies at neutral and acidic pH to gain insight into proton-dependent gating. A 2.6-Å-resolution structure of a resting conformation with a nonconductive TMD was obtained at pH 7.5 (Fig. 2A and figs. S2 to S5). The structure was consistent with the resting structures of the human and pufferfish channels previously determined at lower (3.5 to 3.6 Å) resolution (root mean square deviation = 0.8 Å) (*14*, *15*). A markedly different conformation was obtained at pH 4.5 (Fig. 2B and fig. S2B), at which the channel displays maximum activity (*5*, *10*, *11*). As described below, this 3.1-Å-resolution structure represents an activated conformation of the channel, in part, because it contains an open pore wide enough for hydrated chloride ions to pass through the membrane (Fig. 2, D and F). We observed the activated conformation in both detergent and lipid nanodisc preparations of the channel, indicating that the conformation was independent of the sample preparation method (fig. S3 and Materials and Methods).

ASOR currents diminish upon exposure to pH 4.0, implying that the channel desensitizes, but channel desensitization is less marked at pH ~4.5 (*5*, *11*, *14*). This pH dependence of desensitization and the substantial differences between the pH 4.5 activated structure and the previous nonconductive pH 4.0 structure (*14*) suggest that the pH 4.0 structure represents a desensitized state. Although desensitization is a common theme in the biology of ion channels, a physiological role of ASOR desensitization needs to be established.

Our single-particle cryo-EM data at pH 4.5 revealed populations of channels not only in the activated conformation but also in resting and desensitized conformations (figs. S3, S6, and S7). The detergent-reconstituted sample contained approximately equal fractions of channels in the resting and activated conformations but no substantial fraction of desensitized channels (fig. S6). The lipid nanodisc–reconstituted sample contained all three conformations, with approximate particle distributions of 1 : 1 : 1.8 (resting : activated : desensitized) (fig. S7). A distribution of conducting and nonconducting states at pH 4.5 is consistent with single-channel recordings of the native channel that indicate an open probability of less than 1 at activating pH (*5*). The resting, activated, and desensitized conformations were the only conformations obtained from our cryo-EM analyses, suggesting that they are the primary conformational states of the channel (fig. S3). Well-defined cryo-EM density for each conformation enabled accurate construction of atomic models (figs. S8 to S11 and table S1).

The improved resting and desensitized structures (determined at 2.6 and 2.5 Å resolution, respectively) yielded information regarding the conformations of amino acid side chains (figs. S9 and S11), including those that we identify to be involved in proton dependence. In all three conformational states, the ASOR channel is assembled from a symmetric trimer of TMEM206 subunits and comprises a large ECD (which would be the luminal domain in endosomes), a TMD containing two TM helices per subunit (TM1 and TM2), and an intervening juxtamembrane interface (JMI) that links the ECD to the TMD (Fig. 2, A to C, and fig. S12).

The transition between the resting and activated conformations constitutes an inward contraction of the ECD toward the axis of symmetry and an outward expansion of the upper part of the TMD away from the axis of symmetry (Fig. 2, fig. S13, and movies S1 and S2). This seemingly incongruous transition is enabled by an arrangement, present in both the resting and activated structures, wherein the ECD of one subunit (e.g., the blue-colored ECD in Fig. 2, A and B) is positioned above the TMD of a different subunit (e.g., the red TMD in Fig. 2, A and B). This arrangement arises from a well-structured region of the JMI comprising a four-stranded β sheet in each subunit (the β-sheet coupling domain) that runs roughly parallel to the membrane and connects to the extracellular ends of the TM helices: the β1 strand to TM1 and the β11 strand to TM2 (Fig. 2, A to C). Activation causes the ends of the β-sheet coupling domains of the subunits to move apart from one another (Fig. 2F), which, in turn, results in expansion of the upper part of the TMD.

### Activation creates an ion pore

**Fig. 3.**
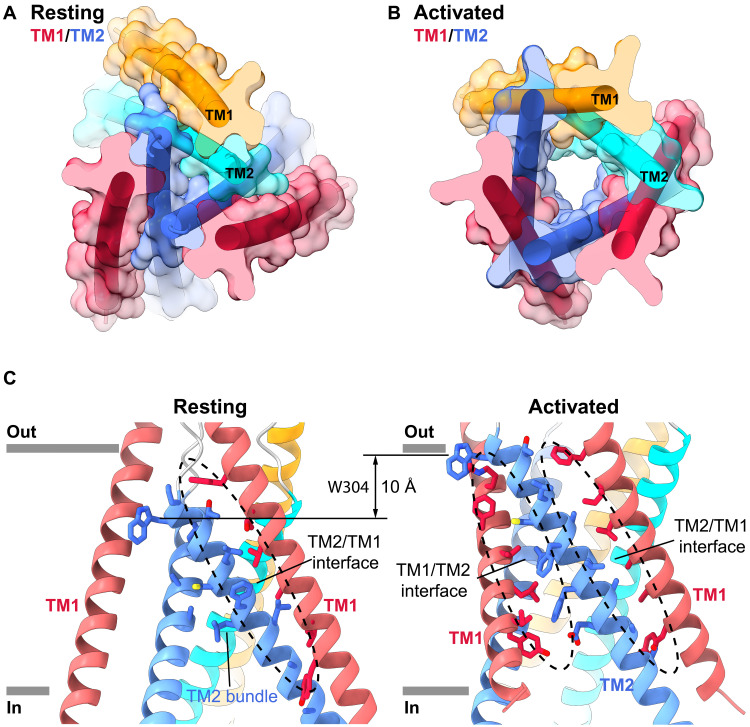
TMD metamorphosis of the activated conformation. (**A** and **B**) TMDs of the resting and activated conformations viewed from the extracellular side, with semitransparent molecular surfaces and α helices as cylinders. For two subunits, TM1 and TM2 are colored red and blue, respectively; for the third subunit, they are colored orange and cyan, respectively. (**C**) TMD interactions in the resting and activated conformations. Interacting residues are drawn as sticks, with interfacial regions indicated by dashed ovals. The displacement of TM1 with respect to TM2 is highlighted by the approximately 10-Å shift of Trp^304^ (W304).

**Fig. 4.**
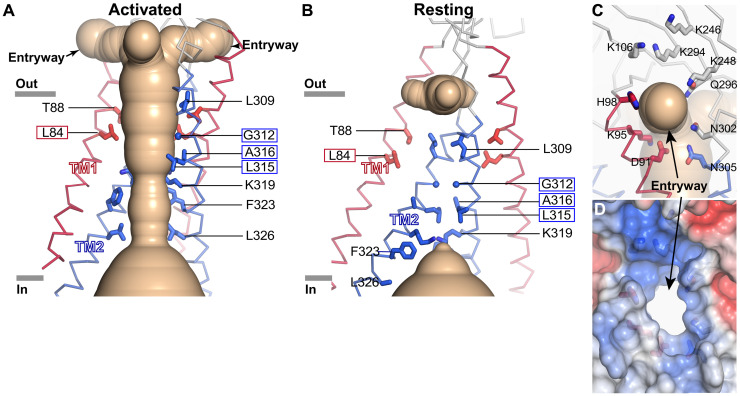
The activated ion pore. (**A**) Ion pore in the activated conformation. The solvent-accessible surface of the pore (wheat color) is depicted, showing the side entryways connecting it with the outside solution. Two subunits of the TMD are shown as ribbons (TM1 helices are red; TM2 helices are blue). Residues that line the walls of the pore are displayed as sticks and are labeled. Boxed labels indicate residues that were accessible to MTSES in activated channels and inaccessible in resting channels (Fig. 5). (**B**) TMD of the resting conformation, depicted as in (A). Narrow dimensions occlude solvent. Amino acids from (A) that form the walls of the activated pore are shown. (**C** and **D**) Close-up views of a side entryway in the activated conformation, showing cartoon (C) and surface-filling (D) representations. The perspective is of the entryway closest to the viewer from (A). In (C), amino acids surrounding the entryway are shown as sticks and the ion pore is depicted as in (A). In (D), the electrostatic surface is shown (red, −5 kT e^−1^; blue, +5 kT e^−1^), with the central white region indicating the opening of the entryway.

Comparisons among TMDs reveal the formation of an ion channel pore in the activated conformation that is absent in the other conformations of the channel. In the resting conformation, a trimeric bundle of tightly packed TM2 helices surrounds the axis of symmetry (Fig. 3A). The TM1 helices are found at the periphery of the TMD, where they pair with TM2 helices from the same subunit (Fig. 3, A and C). The helices do not form an ion conduction pore in this configuration—not only is the central axis, along which the pore resides in the activated channel, too narrow for ion conduction (Fig. 2D) but also the arrangement of the helices does not create an aqueous chamber that is sequestered from the lipid environment through which ions could pass (Fig. 4B). The activated conformation of the pore results from a marked rearrangement of the TM1 and TM2 helices (Fig. 3 and movie S1). The intramembrane reorganization creates an arrangement of three TM1 and three TM2 helices as a barrel with six staves, assembled in an alternating TM1-TM2-TM1 pattern around the axis of symmetry (Fig. 3B). As opposed to the resting structure in which the TM1 helix of a given subunit flanks the TM2 helix of the same subunit, each TM1 now packs between the TM2 helix of its own subunit and TM2 of a neighboring one (Fig. 3, A and B). This molecular rearrangement results in an approximately 50% increase in the interface between the TM helices (1983 Å^2^ resting interface area; 2945 Å^2^ activated interface area; Fig. 3C). In the transition, the TM2 helices are drawn up approximately two helical turns toward the extracellular side with respect to the TM1 helices of the same subunit, completely remodeling the interactions between the helices (Fig. 3C). For instance, TM2 of one subunit, which packed with the TM2 helices of the other two subunits in the resting conformation, now contacts two neighboring TM1 helices but no other TM2 helices (Fig. 3).

The activated arrangement of the TMD creates an aqueous chamber, sealed off from the membrane by the surrounding helices, large enough for hydrated Cl^−^ to permeate (Figs. 2D and 4A). The design is remarkable in its simplicity—one side of each of the TM1 and TM2 helices faces the membrane, and the other contributes to the walls of the ion pore. Cl^−^ ions would not be able to flow through the ECD—its axis of symmetry is too narrow (fig. S13B). Rather, ions would access the pore through three side entryways (fenestrations) in the JMI that are formed in the activated channel (Fig. 4, A, C, and D). The entryways are large enough for hydrated ion permeation in the activated structure, whereas side fenestrations are narrower in the resting and desensitized structures (fig. S14). Formation of the entryways in the activated structure is due, in part, to a compaction of secondary structure in the JMI, wherein a region of the polypeptide preceding TM2 (amino acids 298 to 301) transitions from an extended secondary structure in the resting conformation to an α-helical one (pre-TM2) in the activated conformation (Fig. 2B). The entryways are flanked by hydrophilic and positively charged amino acids that create an environment aptly suited to hydrated anions (Fig. 4, C and D).

### The gate

**Fig. 5.**
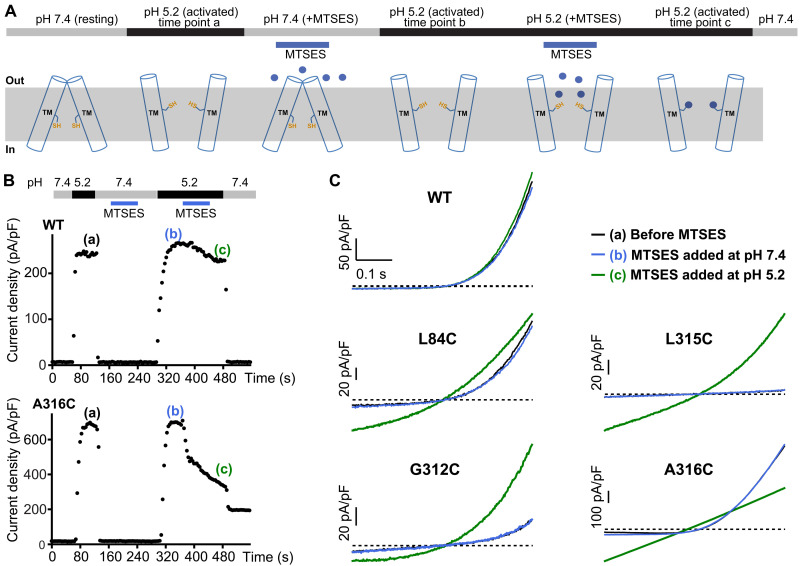
State-dependent accessibilities of cysteine substitutions. (**A**) Schematic of the experiment. The MTSES reagent was applied at the indicated times (blue). (**B**) Representative whole-cell patch-clamp time courses assessed at +80 mV from cells expressing wild-type (WT) or A316C mutant channels. Voltage ramps from −80 to +80 mV were repeatedly applied every 4 s to measure ASOR current. The external pH was controlled as indicated (horizontal bars), with periodic MTSES additions as indicated. MTSES has a negligible effect on A316C channels when added at resting pH 7.4 or on WT channels when added at either pH 7.4 or the activating pH of 5.2. Outward currents through A316C channels are inhibited, however, when MTSES is applied at pH 5.2, and afterward, channels fail to close completely at pH 7.4. (**C**) Representative current traces obtained from −80 to +80 mV voltage ramps for the indicated channels at pH 5.2 before MTSES addition (black, time point “a”), after MTSES application at pH 7.4 (blue, time point “b”), and after MTSES application at pH 5.2 (green, time point “c”).

Changes within the TMD draw one’s attention to a hydrophobic region of TM2 that forms the narrowest constriction in the resting structure (amino acids 308 to 315) and dilates sufficiently for ion permeation in the activated conformation (Fig. 4, A and B), suggesting that it operates as a variable constriction or “gate” that permits or prevents the passage of ions when open or closed. We tested the hypothesis that the narrow constriction of TM2 helices observed in the resting conformation functions in this manner by evaluating the accessibilities of cysteine substitutions within the TMD to the thiol-reactive compound MTSES [(2-sulfonatoethyl) methanethiosulfonate] under resting and activating conditions. MTSES is a water-soluble and membrane-impermeant reagent that has been used previously to map the aqueous pores of ion channels (*21*). Previous studies by the Jentsch group identified that cysteine substitutions of Gly^312^, Leu^315^, and Ala^316^ (on TM2) and Leu^84^ (on TM1) exhibited marked electrophysiological effects when MTSES was applied from the extracellular side, which suggests that these residues line the ion conduction pore and is consistent with the activated structure, but the studies did not determine whether the accessibilities of these residues differed in response to pH-dependent control of the channel (*11*). As in those studies, we used whole-cell patch-clamp electrophysiology to monitor ionic currents in *TMEM206* knockout cells that were transiently expressing wild-type or mutant ASOR (TMEM206) protein. To evaluate the state dependence of the accessibilities to MTSES, the compound was applied from the extracellular side during resting (pH 7.4) or activating (pH 5.2) conditions, and ionic currents were evaluated throughout the experiment (Fig. 5, A and B). Each application of MTSES was followed by washout of the compound. Figure 5C shows current traces elicited by voltage ramps for channels that had been activated by pH 5.2 at three time points during the experiment: (a) before MTSES treatment, (b) following MTSES application at pH 7.4, and (c) following MTSES application at pH 5.2. The wild-type channel displayed indistinguishable current traces at the three time points, indicating that it was not notably affected by MTSES treatment (Fig. 5, B and C), consistent with previous observations (*11*). We found that cysteine substitutions of residues within or below the resting constriction of TM2 (Gly^312^, Leu^315^, and Ala^316^) were unavailable for MTSES modification at pH 7.4 (Fig. 5C and fig. S15), consistent with the absence of an aqueous pore in the resting conformation. At pH 5.2, on the other hand, the cysteine substitutions of Gly^312^, Leu^315^, and Ala^316^ were readily modified by MTSES, as evidenced by marked changes in the current traces (Fig. 5C). In accord with the previous study (*11*), the effects persisted following washout, indicating that the changes were irreversible and consistent with covalent modification by MTSES (Fig. 5B and figs. S15 and S16). The perturbations of the current traces resulting from MTSES modification varied among the residues in interesting ways, but the particulars of these alterations were not the focus of the study, as the primary purpose was to assess whether the residues displayed state-dependent changes in accessibility. The observed modifications of the residues at activating pH are indicative of a wide aqueous pore and consistent with the activated structure. We note that modifications were observed despite generally reduced chemical reactivity of MTSES for solvent-exposed cysteine residues at acidic pH relative to neutral pH, due to protonation of the cysteine thiol. That we do not observe modifications of the residues by MTSES at neutral pH, at which the compound would have greater reactivity for exposed cysteine residues, is an additional indication that the residues are buried in the resting conformation.

Similar state-dependent accessibility of L84C on TM1 was observed (Fig. 5C and fig. S15). This residue, located near the extracellular side of the TMD, is buried in the resting conformation but becomes exposed to the pore in the activated conformation as a result of the TMD rearrangement (Fig. 4, A and B).

The observed reactivity patterns on TM1 and TM2 are beautifully consistent with the resting and activated structures and support the conclusion that they represent physiological states of the channel that are controlled by extracellular pH. Furthermore, the reactivity patterns support the structural observation that the hydrophobic seal made by the Gly^312^, Leu^315^, and Ala^316^ residues of the TM2 helices in the resting structure prevents ion conduction through the channel and thereby functions as a “gate” in a manner of speaking. However, the “gate” should not be ascribed to a few particular residues because metamorphosis of the TMD is responsible for the transition between the nonconductive conformation of the resting structure and the conductive ion pore formed in the activated structure. Gating of ASOR involves not only dilation of a central helical bundle, as is typical for many ion channels, but also complete TM rearrangement. Hence, the entire TMD could be considered the gate of the channel that opens in response to external acidification.

### Proton-sensing mechanism

**Fig. 6.**
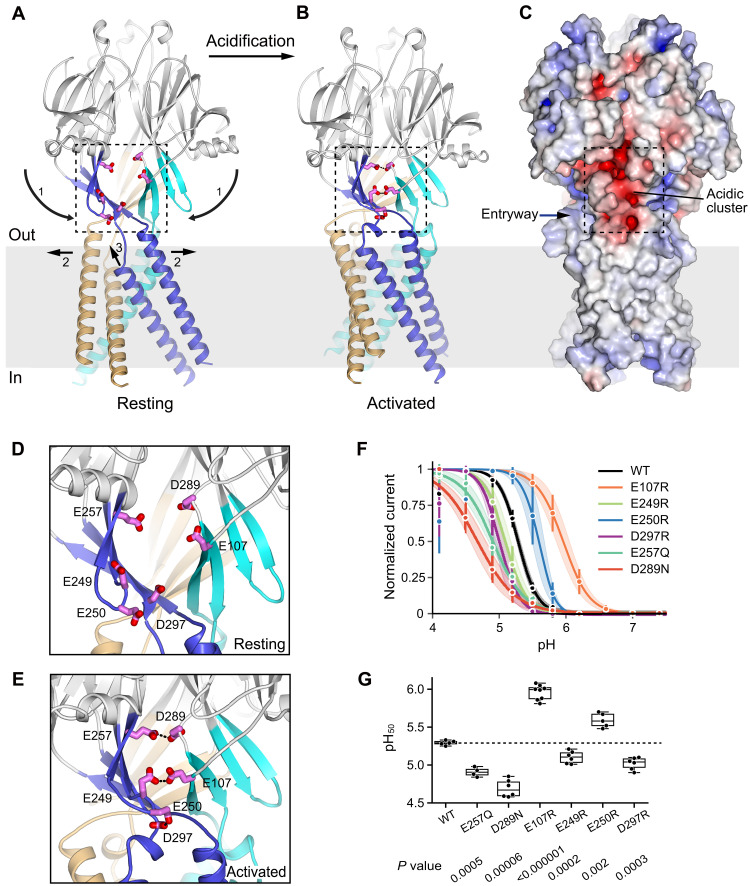
Proton-sensing and gating mechanisms. (**A** and **B**) Resting and activated conformations viewed from the membrane. Each subunit’s JMI and TMD are colored. Movements of the JMI and TMD between the resting and activated states are denoted by arrows. Numbers “1,” “2,” and “3” indicate the conceptual sequence of activation, although the transition may be concerted. The acidic cluster residues at one of the subunit interfaces are shown as magenta sticks. (**C**) Molecular surface of the channel in the activated conformation. Coloring is according to the electrostatic potential, which is contoured from −10 kT e^−1^ (red) to +10 kT e^−1^ (blue). The acidic cluster closest to the viewer is boxed. (**D** and **E**) Close-up views of acidic clusters in resting (D) and activated (E) conformations. The acidic residues (magenta) are shown as sticks and are labeled, with carboxyl-carboxylate pairings indicated by dotted lines. (**F**) pH dependence of current for the WT channel and indicated mutants (whole-cell configuration, at +60 mV, and normalized to maximal values). Dots with error bars represent mean values and SDs; solid lines represent the averaged Boltzmann-fitted data. Degrees of channel desensitization are evident at pH 4.1. Charge reversal E257K and D289K mutants were tested but did not yield measurable currents (fig. S18D). (**G**) Half-maximal pH values obtained from Boltzmann-fitted currents, with boxed regions showing quartiles and medians (independent experiments: *n* = 5 for WT and E250R, *n* = 7 for E257Q, and *n* = 6 for the other constructs). *P* values are indicated (against WT; two-tailed Welch’s *t* test, with false discovery rate controlled by the Benjamini-Hochberg procedure).

Movement in the ECD between the resting and activated structures is characterized by compaction along the symmetry axis and a downward ~14° rotation of each subunit’s ECD (Fig. 6, A and B, and movie S2). This movement brings together three equivalent clusters of six solvent-exposed acidic residues at subunit-subunit interfaces within the JMI (Fig. 6, A to E). These acidic amino acids are part of the β-sheet coupling domain. In the resting conformation, the residues are too far apart to interact with one another (Fig. 6D). In the activated conformation, the amino acids condense (by approximately 7 Å) and pair to form three proton-mediated carboxyl-carboxylate interactions within each cluster (Fig. 6E). Two of the pairings occur between residues on adjacent subunits (Glu^249^ to Glu^107^ and Glu^257^ to Asp^289^), and the third (Glu^250^ to Asp^297^) is between residues of the same subunit (Fig. 6E). Defined cryo-EM density and structure-based estimation of p*K*_a_ values substantiate these interactions and suggest that they are enabled by partial protonation at acidic pH (figs. S10 and S17). Electrophysiological studies show that mutations of these acidic amino acids significantly alter the pH dependence of the channel (Fig. 6, F and G, and fig. S18). As observed for mutations of acidic proton sensors in the ASIC acid-sensing cation channel, the direction of the shift is difficult to predict, as it depends on the local environment and secondary effects of mutations (*20*), but the marked perturbation of pH sensitivity implicates these residues in the proton-sensing mechanism.

### Desensitization

The 2.5-Å-resolution desensitized conformation of the channel obtained at pH 4.5 is analogous to the one previously resolved at 3.7 Å resolution at pH 4.0 (Fig. 2 and fig. S19). The improved resolution allows unambiguous visualization of the amino acid backbone and the side-chain conformations throughout the channel (fig. S11). Density for the acidic clusters, JMI, and TMD regions is particularly improved (fig. S19). The ECD of the desensitized structure adopts a similar conformation as in the activated one, complete with the formation of carboxyl-carboxylate pairings of amino acids in the acidic clusters (Fig. 2 and figs. S20A and S21). The TMD of the desensitized conformation, on the other hand, is different from that of both the resting and activated structures (Fig. 2 and fig. S20). The ion conduction pore observed in the activated structure is no longer present (Fig. 2D and fig. S20, B and C). Instead, the TM2 helices pack in trimeric fashion, with interactions among them similar to the resting conformation that seal off the ion pore (fig. S22). The TM1 helices are located at the periphery of the TMD and are repositioned from both their resting and activated configurations. They primarily interact with the TM2 helices of a neighboring subunit, rather than with their own as in the resting conformation (fig. S20B), and instead of pointing down and to the right, each TM1 is oriented down and to the left in the desensitized conformation (Fig. 2C). Changes in the JMI-TMD linkers accompany the desensitized transformation of the TMD (Fig. 2 and fig. S20A).

On the basis of the previous structure of the desensitized conformation, it was proposed that the side chain of His^98^, although not well defined in the map, might bind within the acidic cluster and be an integral part of the channel’s proton dependence (*14*). The side chain of His^98^ is not positioned within the acidic cluster in any of our structures, and it is too far to make direct contacts with any of the acidic amino acids (fig. S21). It has been shown that mutation of His^98^ has only a modest effect on the pH dependence of the channel (*14*). Accordingly, structure-based assessments of the p*K*_a_ of His^98^ indicate only slight perturbations from canonical values for histidine (fig. S17C). We conclude that His^98^ is not a primary component of the proton activation mechanism.

### Distinct activation mechanism of ASOR

Our data support the conclusion that the acidic clusters function as the primary proton sensors and drive the conformational changes that lead to channel activation in the scheme shown in Fig. 6 (A and B). At neutral resting pH, the acidic amino acids do not interact with one another and no ion permeation path through the membrane exists. Collapse of the acidic clusters at activating low pH causes inward rotation and compaction of the ECD. The β-sheet coupling domains act as push devices to transmit this compaction into dilation of the upper TMD. This causes reorganization of the TM helices into an ion conduction pathway in the activated state. The mechanics of the activation process thereby transform rigid body motions within the ECD into TMD metamorphosis.

We find that the proton-sensing mechanism of ASOR has conceptual similarities to the ASIC family of acid-sensing cation channels (*19*) but that the gating mechanism of the ion pore is markedly distinct. ASIC channels have extracellular acidic pockets that are implicated in proton sensing and coalesce at low pH (fig. S23) (*16*, *18*, *19*). These acidic pockets are in the upper region of ASIC’s ECD, rather than in the lower ECD as in ASOR, and are located on an α-helical region not found in ASOR (fig. S23). In ASIC, the pockets are composed of amino acids within the same subunit and induce intrasubunit conformational changes at low pH that propagate to the TMD to open the pore (fig. S23) (*18*, *19*). Unlike the substantial TMD rearrangements in ASOR, pore opening in ASIC occurs through a modest widening of the pore through iris-like movements of its TM2 helices, which form the walls of its pore in both the resting and activated conformations. Like ASOR, the ASIC family also desensitizes (*17*). However, unlike ASOR, the desensitized TMD of ASIC is structurally analogous to its resting state (*22*). A region of the ECD in ASIC channels acts as a “molecular clutch” to allow partial decoupling between the ECD and the TMD in desensitized channels (*19*, *22*). In ASOR, the desensitized TMD is markedly altered from the resting and activated conformations, suggesting that the TMD itself, together with modest changes in the JMI, acts as a molecular clutch. Our work defines principles of proton sensing in ASOR and reveals pH-dependent TM metamorphosis that represents a new paradigm of ion channel gating.

## MATERIALS AND METHODS

### Protein expression and purification

Complementary DNA (cDNA) encoding full-length human ASOR (TMEM206; UniProt: Q9H813) was codon-optimized, synthesized (IDT Inc.), and ligated into a mammalian cell expression vector (*23*) to encode a protein containing a C-terminal green fluorescent protein (GFP) tag, which could be removed using PreScission protease. The expression plasmid was transfected into Expi293F cells (Invitrogen, #A14527) for transient expression. Briefly, 1 mg of plasmid and 3 mg of PEI25k (Polysciences Inc.) were mixed in 100 ml of Opti-MEM medium (Invitrogen) and incubated at room temperature for 20 min, and the mixture was added into 1 liter of Expi293 cells (3.0 × 10^6^ to 3.5 × 10^6^ cells/ml) in Expi293 expression medium (Invitrogen). After incubation at 37°C for 16 hours, 10 mM sodium butyrate (Sigma-Aldrich) was added, and the cells were cultured at 30°C for another 48 to 72 hours before harvesting.

For protein purification, the cell pellet from 1 liter of culture was resuspended in 100 ml of lysis buffer [40 mM Hepes (pH 7.5), 200 mM NaCl, deoxyribonuclease I (0.15 mg/ml; Sigma-Aldrich), leupeptin (1.5 μg/ml; Sigma-Aldrich), pepstatin A (1.5 μg/ml; Sigma-Aldrich), 1 mM 4-(2-aminoethyl)benzenesulfonyl fluoride hydrochloride (Gold Biotechnology), 1 mM benzamidine (Sigma-Aldrich), 1 mM phenylmethylsulfonyl fluoride (Acros Organics), and 1:500 dilution of aprotinin (Sigma-Aldrich)], then solubilized by adding 0.5% (w/v) digitonin (Cayman Chemical Company), and stirred at 4°C for 1 hour. Solubilized proteins were separated from the insoluble fraction by centrifugation at 60,000*g* at 4°C for 1 hour, and the supernatant was filtered through a 0.22-μm polystyrene membrane (Millipore). The ASOR-GFP fusion protein was purified by affinity chromatography using a nanobody against GFP (*24*). The nanobody was coupled to CNBr-activated Sepharose Fast Flow resin (GE Healthcare) according to the manufacturer’s protocol. Resin (2 ml) was incubated with the sample with agitation at 4°C for 1 hour. The beads were washed with 100 ml of buffer A [20 mM Hepes (pH 7.5), 150 mM NaCl, and 0.05% digitonin], and purified ASOR protein was eluted by adding PreScission protease (~0.1 mg, without additional reducing agent) and incubating for 3 hours at 4°C. The sample was further purified by size exclusion chromatography (SEC; using a Superose 6 increase, 10/300 GL column, GE Healthcare) in gel filtration buffer [5 mM Hepes (pH 7.5) and 150 mM NaCl] containing either 0.02% digitonin or 0.02% glyco-diosgenin (GDN; Anatrace). The peak fractions were pooled and concentrated to 3.5 to 5 mg/ml using a 100-kDa concentrator and immediately used to prepare cryo-EM grids.

### Lipid nanodisc reconstitution

The scaffold protein saposin A was used for lipid nanodisc preparations because of its stability at both neutral and acidic pH (*25*, *26*). Saposin A was expressed and purified according to a published protocol (*27*). On-bead reconstitution of ASOR into saposin-based nanodiscs was modified from a previously published protocol (*28*). Two milliliters of GFP nanobody resin containing approximately 0.5 mg of bound ASOR-GFP was mixed with 320 μl of a lipid/detergent mixture [17 mM dodecyl maltoside (Anatrace), 5 mM 1-palmitoyl-2-oleoyl-sn-glycero-3-phosphocholine (POPE; Avanti), and 5 mM total brain lipids (Avanti)] and combined with 2.8 ml of saposin A (0.17 mM final concentration). After a 10-min incubation at room temperature with agitation, ~1.5 g of wet Bio-Beads SM2 (Bio-Rad) was added to the resin, and the sample was rotated at 4°C for ~16 hours to remove detergent. The resin/Bio-Bead mixture was transferred into a column and washed with 100 ml of buffer [20 mM Hepes (pH 7.5) and 300 mM NaCl] to remove empty saposin nanodiscs. Channel-nanodisc complexes were then eluted by adding PreScission protease (~0.1 mg) and incubating for 3 hours at 4°C. The sample was further purified by SEC (using a Superose 6 increase, 10/300 GL column, GE Healthcare) in gel filtration buffer [5 mM Hepes (pH 7.5) and 150 mM NaCl]. The sample was then concentrated to 8 mg/ml using a 100-kDa concentrator (Vivaspin-2) and immediately used to prepare cryo-EM grids.

### EM sample preparation and data acquisition

For cryo-EM studies at pH 7.5, 4 μl of purified sample was applied to glow-discharged (10 s) Quantifoil R 1.2/1.3 grids (Au 400; Electron Microscopy Sciences) and plunge-frozen in liquid nitrogen–cooled liquid ethane, using Vitrobot Mark IV (FEI), operated at 4°C, with a blotting time of 2 to 3 s, using a blot force of “0,” and 100% humidity. To obtain the samples at pH 4.5, 0.4 μl of 500 mM sodium citrate (pH 4.0) was added to 3.6 μl of protein sample and incubated for 2 to 5 min before freezing (a final pH of 4.5 was measured using a pH meter with mock solutions and confirmed using pH paper). For the samples in saposin nanodiscs, 1.5 mM fluorinated fos-choline-8 (Anatrace) was added to the sample to minimize preferred orientations. Micrographs were collected using a Titan Krios microscope (Thermo Fisher Scientific) operated at 300 kV using a K3 Summit detector [Gatan, at Memorial Sloan Kettering Cancer Center (MSKCC)] or a K3 Summit detector with a GIF quantum energy filter [20 eV, at the Pacific Northwest Center for Cryo-EM (PNCC)] in superresolution mode. Details of all datasets are summarized in table S1. All datasets were processed using the same general workflow as outlined below.

### Cryo-EM structure determination

Figures S5 to S7 show cryo-EM workflows. Image processing was performed in RELION 3.1 (*29*) and cryoSPARC v.2 (*30*). Movie stacks were gain-corrected, twofold binned, motion-corrected, and dose-weighted using MotionCor2 (*31*). Contrast transfer function (CTF) estimates were performed in GCTF (*32*), and micrographs with a CTF fit resolution better than 5 Å were selected. Particles were autopicked and extracted using RELION 3.1 and then imported into cryoSPARC v.2 for further processing. Global and local resolution estimates were determined in cryoSPARC v.2 (*30*).

For the dataset at pH 7.5 in detergent (digitonin), 4,027,178 particles were extracted from 3450 micrographs in RELION 3.1 with a bin factor of 3 and imported into cryoSPARC v.2 for ab initio reconstruction and heterogeneous refinement. The particles were subjected to one round of heterogeneous refinement [three-dimensional (3D) classification] in cryoSPARC v.2 to remove erroneously picked particles that did not resemble the channel. Selected particles (612,580) were used for several rounds of heterogeneous refinement (using C1 symmetry) to remove those that did not yield high-resolution reconstructions. We did not observe evidence of alternative conformations of the channel in the dataset (e.g., from heterogeneous refinement). After the heterogeneous refinement procedure, 152,618 particles were selected, reextracted, and subjected to nonuniform refinement in cryoSPARC v.2 with C3 symmetry, which yielded a reconstruction at 3.0 Å overall resolution. After two rounds of Bayesian polishing in RELION 3.1, the particles were further classified by 3D classification with C1 symmetry in RELION 3.1. The particles selected from the highest-resolution class (60,374) were then subjected to CTF (both global and local) and nonuniform refinements in cryoSPARC v.2, resulting in a map at 2.6 Å overall resolution (map 1). To optimize density for acidic amino acids in the acidic clusters, two rounds of heterogeneous refinement were performed in cryoSPARC v.2 focused on the acidic clusters. 3D reconstruction of the selected and aligned particles (17,681) resulted in a map at 2.7 Å overall resolution (map 2). The final map of the resting conformation was obtained by combining maps 1 and 2 in Chimera (*33*) and was used for subsequent model building and analysis. The cryo-EM workflows for other datasets at pH 7.5 (in GDN detergent or saposin lipid nanodiscs) followed similar procedures (fig. S5) and yielded reconstructions of the resting conformation at 2.9 and 3.4 Å, respectively.

For the dataset at pH 4.5 in GDN detergent, 4,834,754 particles were extracted from 4211 micrographs in RELION 3.1 with a bin factor of 3 and imported into cryoSPARC v.2 for further processing. The particles were subjected to one round of heterogeneous refinement in cryoSPARC v.2 to remove erroneously picked particles that did not resemble the channel. Selected particles (1,044,859) were used for ab initio reconstructions and heterogeneous refinements, and this yielded both activated and resting conformations of the channel. Particles from the activated (379,992 particles) and resting (342,939 particles) classes were subsequently processed separately. Particles from the activated class were subjected to several rounds of heterogeneous refinement, from which 119,911 particles were chosen, reextracted without binning, and subjected to nonuniform refinement, which yielded a reconstruction at approximately 3.1 Å overall resolution. To optimize the density in the TMD, the aligned particles were imported into RELION 3.1 for signal subtraction (retaining only the TMD) and 3D classification without alignment. Particles (36,051) from the class with the most well-defined density in the TMD were imported into cryoSPARC v.2 for nonuniform refinement, followed by two rounds of Bayesian polishing in RELION 3.1 and CTF refinement (both global and local) in cryoSPARC v.2, which yielded a reconstruction at 3.1 Å overall resolution, but with improved density in the TMD (map 1). To optimize the density of acidic residues, low-dose images (3 e/Å^2^, representing the first six of movie frames) were used to reconstruct a map in RELION 3.1 (map 2). The final map of the activated conformation was obtained by combining maps 1 and 2 in Chimera and was used for subsequent model building and analysis.

Particles from the resting class obtained at pH 4.5 in GDN detergent were subjected to several rounds of heterogeneous refinement, from which 63,587 particles were chosen, reextracted without binning, and subjected to nonuniform refinement, which yielded a reconstruction at 3.5 Å resolution at this stage. After one round of Bayesian polishing and 3D classification in RELION 3.1, 36,051 particles were selected and subjected to CTF (both global and local) and nonuniform refinements in cryoSPARC v.2, resulting in a map at 3.2 Å overall resolution.

For the dataset at pH 4.5 in saposin lipid nanodiscs, 12,000,056 particles were extracted from 14,606 micrographs in RELION 3.1 with a bin factor of 3 and imported into cryoSPARC v.2 for further processing. The particles were subjected to one round of heterogeneous refinement in cryoSPARC v.2 to remove erroneously picked particles that did not resemble the channel. The resulting 3,097,510 particles were selected for ab initio reconstruction and heterogeneous refinement, yielding resting, activated, and desensitized conformations of the channel, representing 22, 22, and 39% of the particles, respectively. No additional conformations of the channel could be discerned from the analysis. The particles from the resting and activated classes were subjected to several rounds of heterogeneous refinements. From these, 109,714 particles in resting class and 43,714 particles in activated class were selected, reextracted, and used for nonuniform refinement to yield 3.2- and 3.5-Å reconstructions, respectively. The particles from the desensitized class were subjected to several rounds of heterogeneous refinement, from which 414,821 particles were selected, reextracted, and used for nonuniform refinement, yielding a reconstruction in the desensitized conformation at 2.8 Å resolution. Following two rounds of Bayesian polishing and 3D classification in RELION 3.1, selected particles (157,906) were then subjected to CTF refinement (both global and local) and nonuniform refinement in cryoSPARC v.2. This yielded the final map of the desensitized conformation at 2.5 Å overall resolution.

Atomic models were built de novo, refined in real space using COOT (*34*), and further refined in real space using PHENIX (*35*). The final models have good stereochemistry and good Fourier shell correlations with the cryo-EM map (figs. S8 to S11 and table S1). Structural figures were prepared with PyMOL (pymol.org) (*36*), Chimera (*33*), ChimeraX (*37*), CAVER (*38*), and HOLE (*39*). PROPKA 3.0 (*40*) was used to estimate p*K*_a_ values from the structures. Buried surface areas were calculated using PyMOL by taking the difference between the cumulative molecular surface area of the three individual subunits and the molecular surface of the assembled trimer (using a solvent radius of 1.4 Å).

### Anion flux assay

The channel was purified as described for cryo-EM analysis, using slightly different detergents that allow more facile dialysis: *n*-dodecyl-β-d-maltopyranoside for extraction and *n*-decyl-β-d-maltopyranoside (3 mM) during gel filtration. The liposome reconstitution procedure was adapted from previous protocols (*41*–*44*). Briefly, a 3:1 (w:w) mixture of POPE (Avanti) and POPG (1-palmitoyl-2-oleoyl-sn-glycero-3-phospho-(1′-rac-glycerol) (Avanti) lipids was prepared at 20 mg/ml in reconstitution buffer [100 mM potassium sulfate and 4 mM MES-NaOH (pH 6.0)], and the lipids were solubilized by adding 8% *n*-octyl-β-d-maltopyranoside (w/v) (Anatrace). The protein was then combined with an equal volume of the solubilized lipids to give a final protein concentration of 0.1 mg/ml and a lipid concentration of 10 mg/ml. Detergent was removed by dialysis (8000 Da molecular weight cutoff) at 4°C against a total of 10 liters of reconstitution buffer with daily buffer exchanges over the course of 5 days. Empty liposomes (without protein) were prepared in parallel in the same manner using purification buffer instead of purified protein. Following dialysis, the liposomes were sonicated for approximately 20 s in a water bath, divided into aliquots, and flash-frozen in liquid nitrogen for storage at −80°C.

The flux assay was based on previously published methods (*42*). Vesicles were thawed in a 37°C water bath, sonicated (for ∼20 s, in 10-s intervals), and incubated at room temperature for 2 hours. The flux assay buffer consisted of 4 mM MES-NaOH (pH 6.0), bovine serum albumin (0.5 mg/ml), 1.5 μM 9-amino-6-chloro-2-methoxyacridine [ACMA; Sigma-Aldrich, from a 2 mM stock solution in dimethyl sulfoxide (DMSO)], and either 125 mM NaCl, 125 mM NaBr, 125 mM NaNO_3_, or 125 mM NaGlutamate, depending on the anion being tested. Data were collected on a SpectraMax M5 fluorometer (Molecular Devices) using the SoftMax Pro 7 software package. Fluorescence intensity measurements were collected every 30 s with excitation and emission wavelengths of 410 and 490 nm, respectively.

The course of the experiment was as follows: The fluorescence of the flux assay buffer (1 ml) was recorded for 120 s, liposomes were added by 50-fold dilution, and 1 μM of the proton ionophore carbonyl cyanide *m*-chlorophenyl hydrazone (CCCP; Sigma-Aldrich; from a 1 mM stock solution in DMSO) was added at the 300-s time point. The sample was gently mixed with a pipette after each addition. To confirm that the vesicles were intact and to establish as minimum fluorescence baseline, 2 nM valinomycin (Sigma-Aldrich; from a 2 μM stock solution in DMSO) was added after the 1260-s time point (valinomycin allows selective K^+^ efflux from the vesicles, creating a negative charge within them that is used to drive the uptake of H^+^).

### Electrophysiology

*TMEM206^−/−^* HEK293 cells (*11*) were maintained in Dulbecco’s modified Eagle’s medium (PAN Biotech) supplemented with 10% fetal bovine serum (PAN Biotech) and 1% penicillin/streptomycin at 37°C and 5% CO_2_. Cells were transfected using FuGENE HD (Promega, for acidic cluster mutants) or Lipofectamine 2000 (Invitrogen, for cysteine substitution experiments) transfection reagent according to the manufacturers’ instructions 18 to 24 hours before recording. Immediately before recording, cells were plated onto poly-l-lysine–coated coverslips. For mutations of acidic cluster residues, *TMEM206* variants were inserted into pIRES2-EGFP (enhanced GFP) (Clontech). For cysteine substitution experiments, the pcDNA 3.1(+) vector (Invitrogen) was used and *TMEM206* variants were coexpressed with pEGFP-N1 plasmid (Clontech). Point mutations were introduced by QuikChange (Agilent) and validated by Sanger sequencing. Transfected cells were detected by the fluorescence of EGFP.

Whole-cell patch-clamp electrophysiology was performed using a MultiClamp 700B patch-clamp amplifier/Digidata 1550B digitizer and pClamp 10 software (Molecular Devices) with a sampling rate of 5 kHz and a low-pass filter at 4 kHz (pH dependence measurements) or using an EPC-10 USB patch-clamp amplifier and PatchMaster software (HEKA Elektronik) with a sampling rate of 1 kHz and a low-pass filter at 10 kHz (MTSES measurements). Patch pipettes had a resistance of 2 to 6 megohms. Series resistance did not exceed 10 megohms, and series resistance was compensated for by at least 30%.

Patch pipette solution contained 140 mM CsCl, 5 mM EGTA, 1 mM MgCl_2_, and 10 mM Hepes, pH adjusted to 7.2 with CsOH (275 mOsm/kg). Standard bath solution contained 150 mM NaCl, 6 mM KCl, 1 mM MgCl_2_, 1.5 mM CaCl_2_, 10 mM glucose, and 10 mM Hepes (pH 7.4) (NaOH). Bath solutions with pH 6.2 to 5.5 and 5.2 to 4.1 were buffered with 10 mM MES or 5 mM sodium citrate, respectively, and contained CsCl instead of KCl (320 mOsm/kg). Osmolalities of all solutions were measured with an Osmomat 030 freezing point osmometer (Gonotec). Liquid junction potentials were not corrected for. All experiments were performed at room temperature.

After membrane rupture, voltage was held at −30 mV. The protocol to measure pH dependence of *I*_ASOR_ was applied every 5 s and consisted of a 100-ms voltage step to −60 mV, followed by a 2-s voltage ramp from −60 to +60 mV and another 100-ms voltage step at +60 mV. The protocol to assess the effects of MTSES on *I*_ASOR_ was applied every 4 s and consisted of a 100-ms voltage step to −80 mV, followed by a 500-ms voltage ramp from −80 to +80 mV. Current values were normalized to cell capacitance.

MTSES (Biotium) powder was stored at −20°C, and fresh stocks (250 mM in water) were prepared before recording, stored frozen, and thawed to prepare the MTSES-containing bath solutions every 1.5 hours. pH sensitivity was assessed with a protocol that consisted of sequential steps to progressively acidic pH values alternated by periods at pH 7.4. Background current at pH 7.4 was subtracted from the other pH steps. Maximal current amplitudes at each pH value were normalized to the maximal value across the recording and fitted with the Boltzmann equation to obtain the pH_50_ values (the pH value at which current reaches the half of its maximal amplitude). Rundown (desensitization) at extreme acidic pH values may cause underestimation of the maximal value and slight overestimation of pH_50_ values. To mitigate this effect, cells that showed rundown at a pH higher than (pH_50_ – 1) were excluded from analysis. Data analysis was performed using SciPy 1.5.2 library for Python 3.8 programming language (Python Software Foundation).
