# Supplementary material for: Gating choreography and mechanism of the human proton-activated chloride channel ASOR
Source: Sci Adv. 2022 Feb 2;8(5):eabm3942. doi: 10.1126/sciadv.abm3942 (PMC8809534; doi:10.1126/sciadv.abm3942)
Supplement: Supplementary file 1 — Figs. S1 to S23 Table S1 Legends for movies S1 and S2 [file sciadv.abm3942_sm.pdf]

Science Advances
AAAS

Supplementary Materials for

Gating choreography and mechanism of the human proton-activated chloride channel ASOR

Chongyuan Wang, Maya M. Polovitskaya, Bryce D. Delgado,
Thomas J. Jentsch*, Stephen B. Long*

*Corresponding author. Email: longs@mskcc.org (S.B.L.); jentsch@fmp-berlin.de (T.J.J.)

Published 2 February 2022, *Sci. Adv.* **8**, eabm3942 (2022)
DOI: 10.1126/sciadv.abm3942

The PDF file includes:

Figs. S1 to S23
Table S1
Legends for movies S1 and S2

Other Supplementary Material for this manuscript includes the following:

Movies S1 and S2

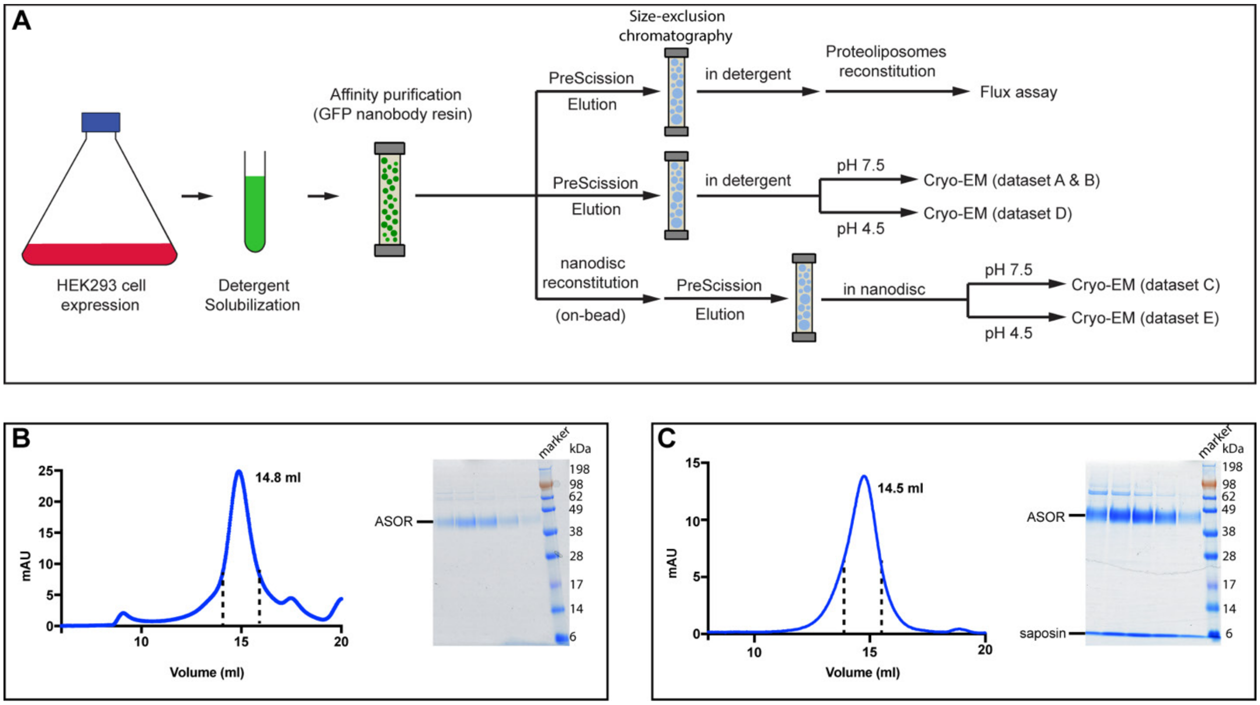

Fig. S1. ASOR expression and purification. (**A**) Purification scheme. (**B**) Representative SEC profile and SDS-PAGE analysis of purified ASOR in detergent. (**C**) Representative SEC profile and SDS-PAGE analysis of the sample in saposin nanodiscs.

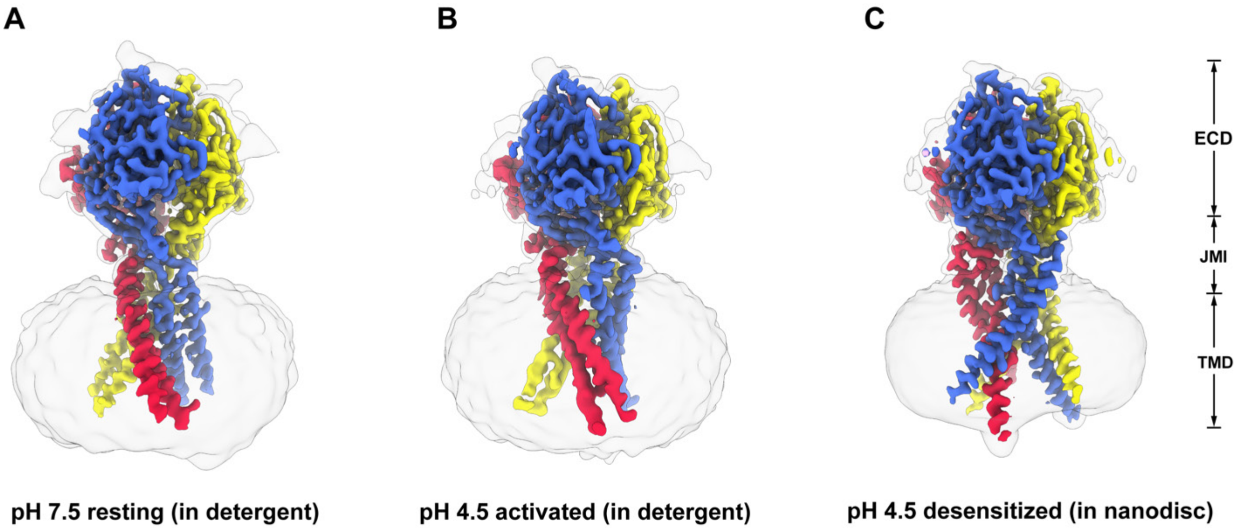

Fig. S2. Cryo-EM maps of the resting, activated, and desensitized conformations. (**A–C**) Maps of the structures. Density for each subunit is colored separately, as in Figure 2. A lower contour is shown as a semi-transparent gray surface to highlight the membrane-spanning region.

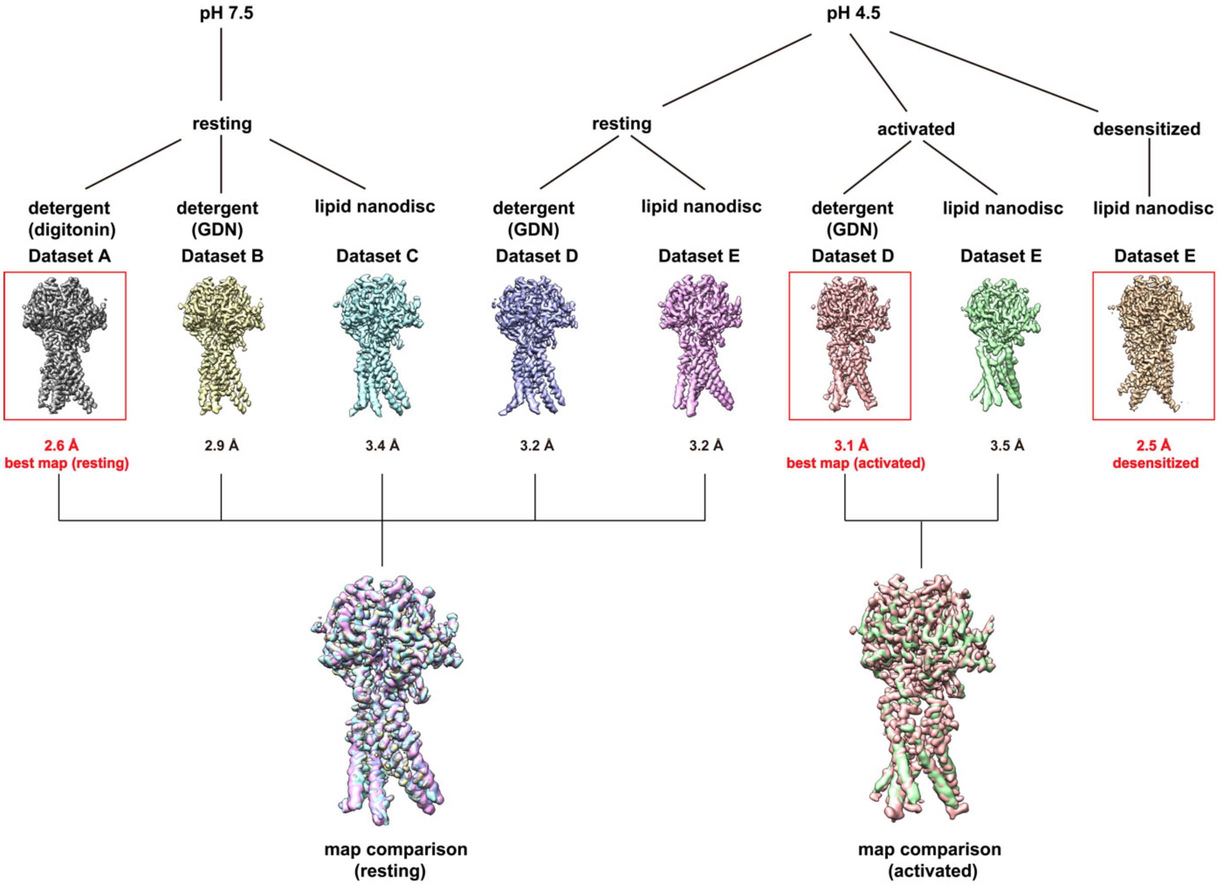

Fig. S3. Cryo-EM reconstructions. Five cryo-EM datasets (denoted datasets A–E) of the channel in detergent (digitonin or GDN) or saposin lipid nanodisc preparations at pH 7.5 and pH 4.5 yielded eight cryo-EM reconstructions. These corresponded to three conformations of the channel (resting, activated, and desensitized), as indicated. The highest resolution structure obtained for each is denoted by a red box. Comparisons among reconstructions obtained for the same conformation are shown in lower panels. The mean correlation coefficients for these groups are 0.88 and 0.85 (resting and activated, respectively, calculated using Chimera (*33*)).

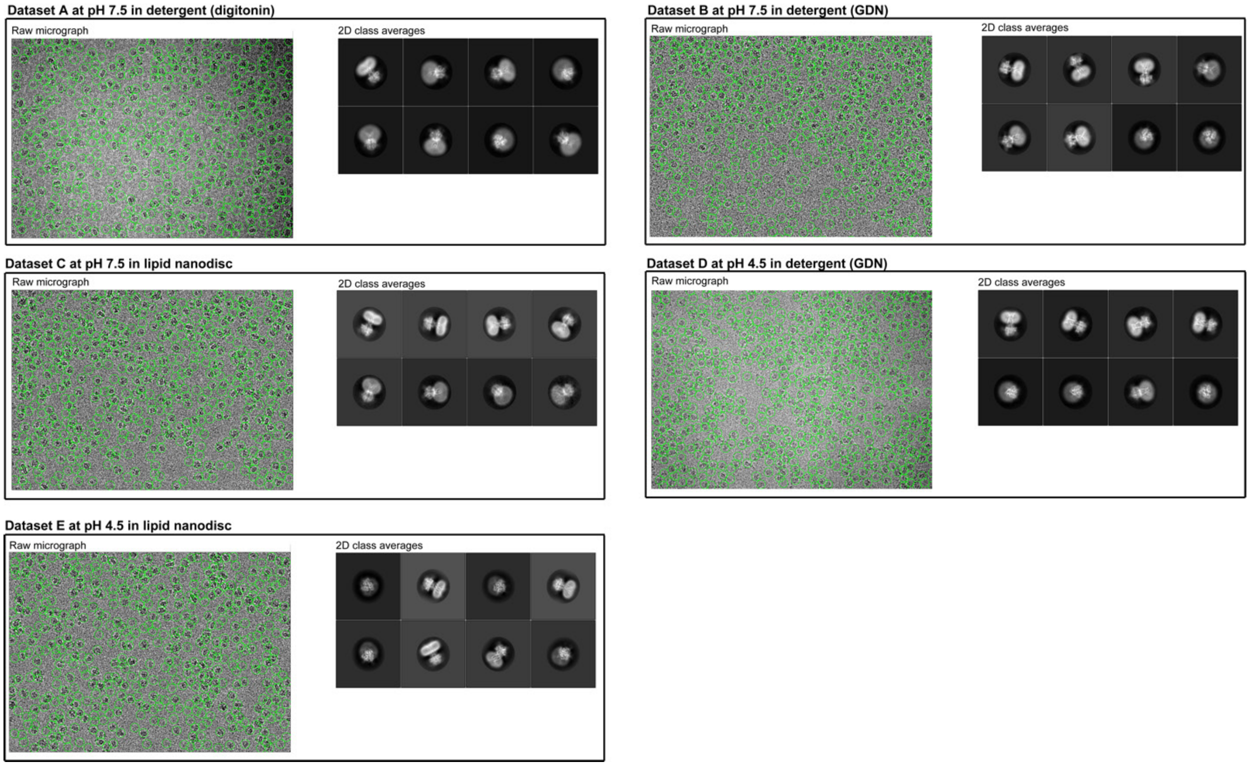

Fig. S4. Representative cryo-EM micrographs and 2D class averages from the datasets. Picked particles are circled (left). Representative 2D class averages of particles are shown (right).

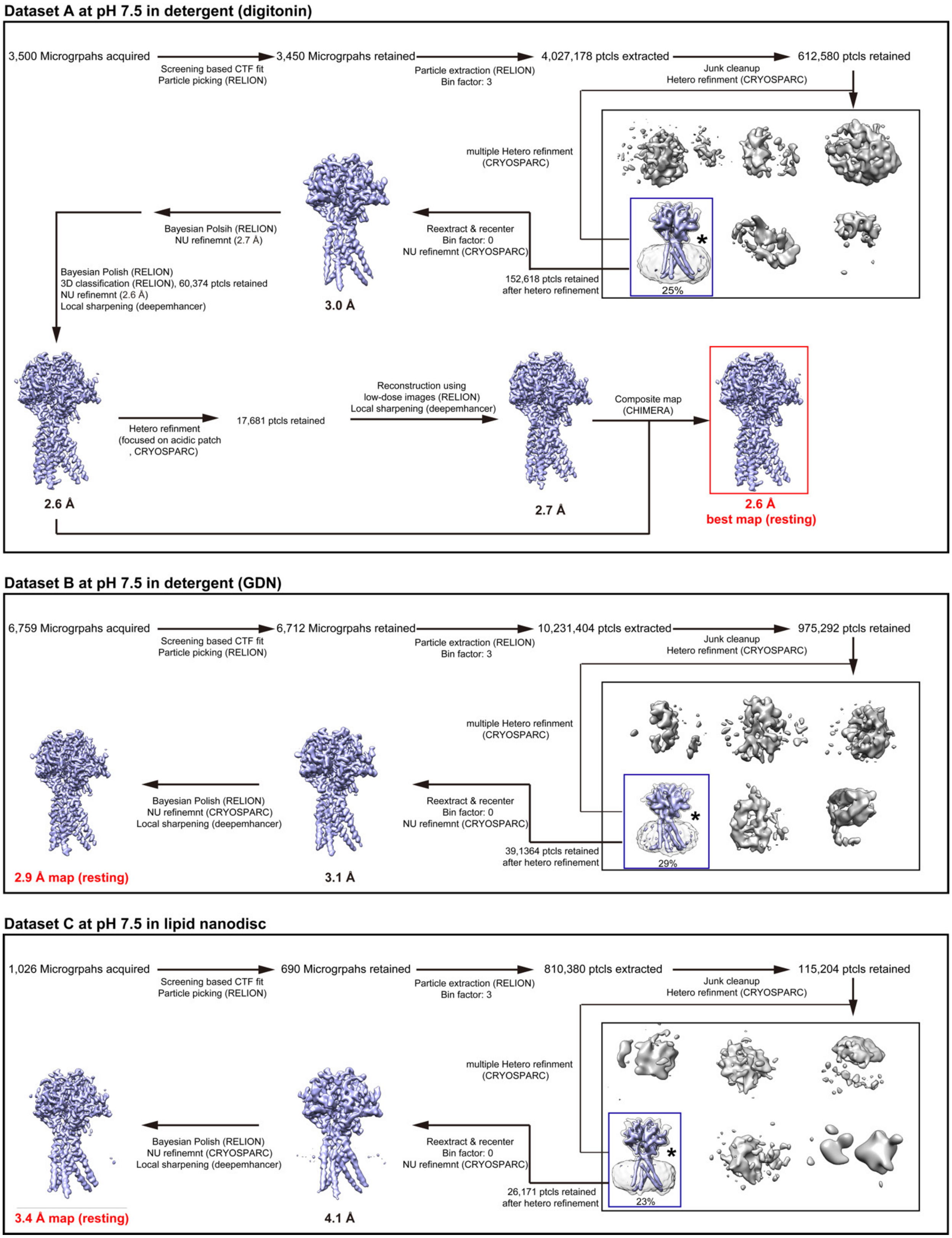

Fig. S5. Flow charts outlining the cryo-EM processing workflows of datasets collected at pH 7.5 (Datasets A - C). Details can be found in Methods.

Dataset D at pH 4.5 in detergent (GDN)

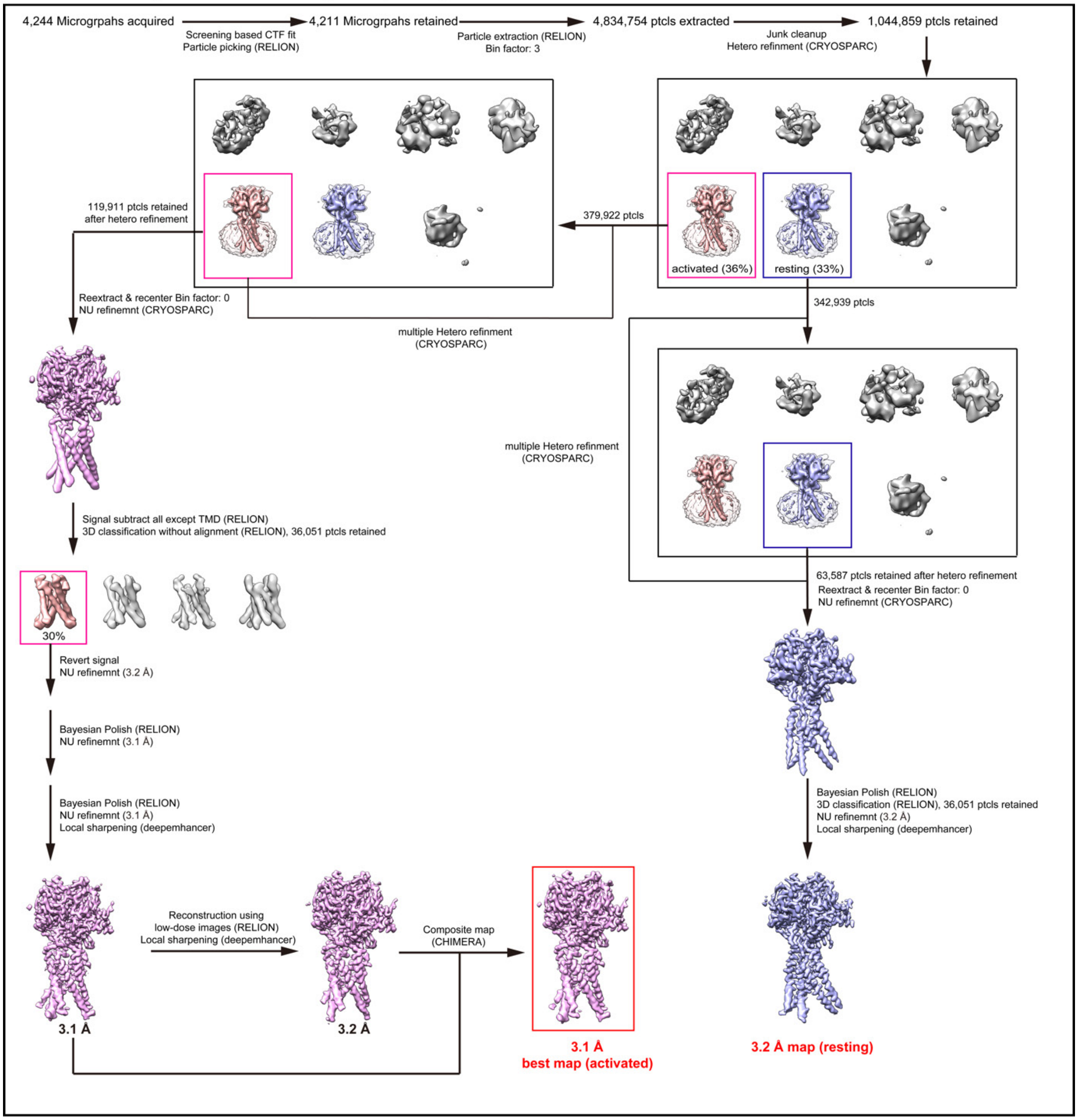

Fig. S6. Flow chart outlining the cryo-EM processing workflow of Dataset D (GDN detergent at pH 4.5). Details can be found in Methods.

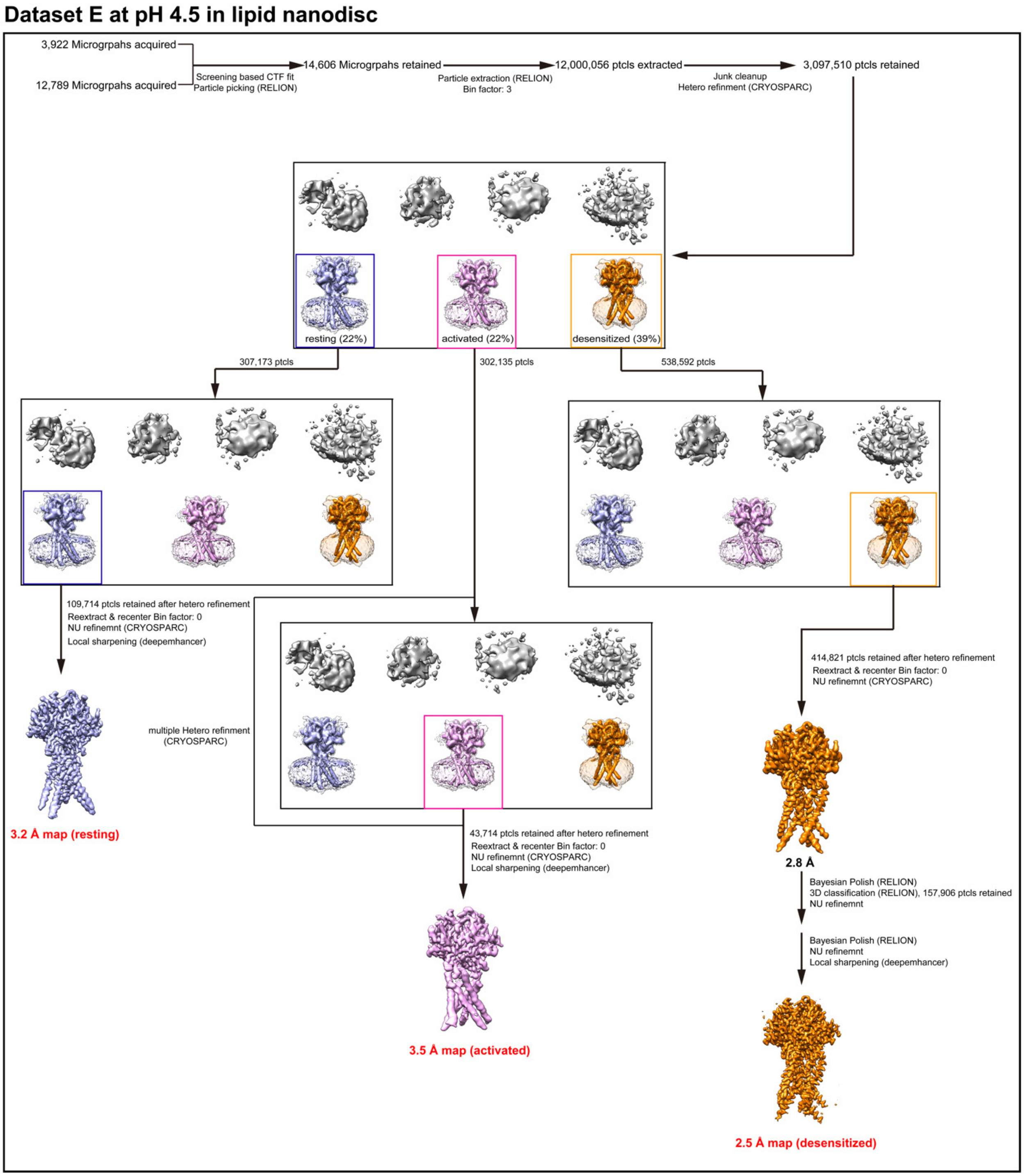

Fig. S7. Flow chart outlining the cryo-EM processing workflow of Dataset E (saposin lipid nanodiscs at pH 4.5). Details can be found in Methods.

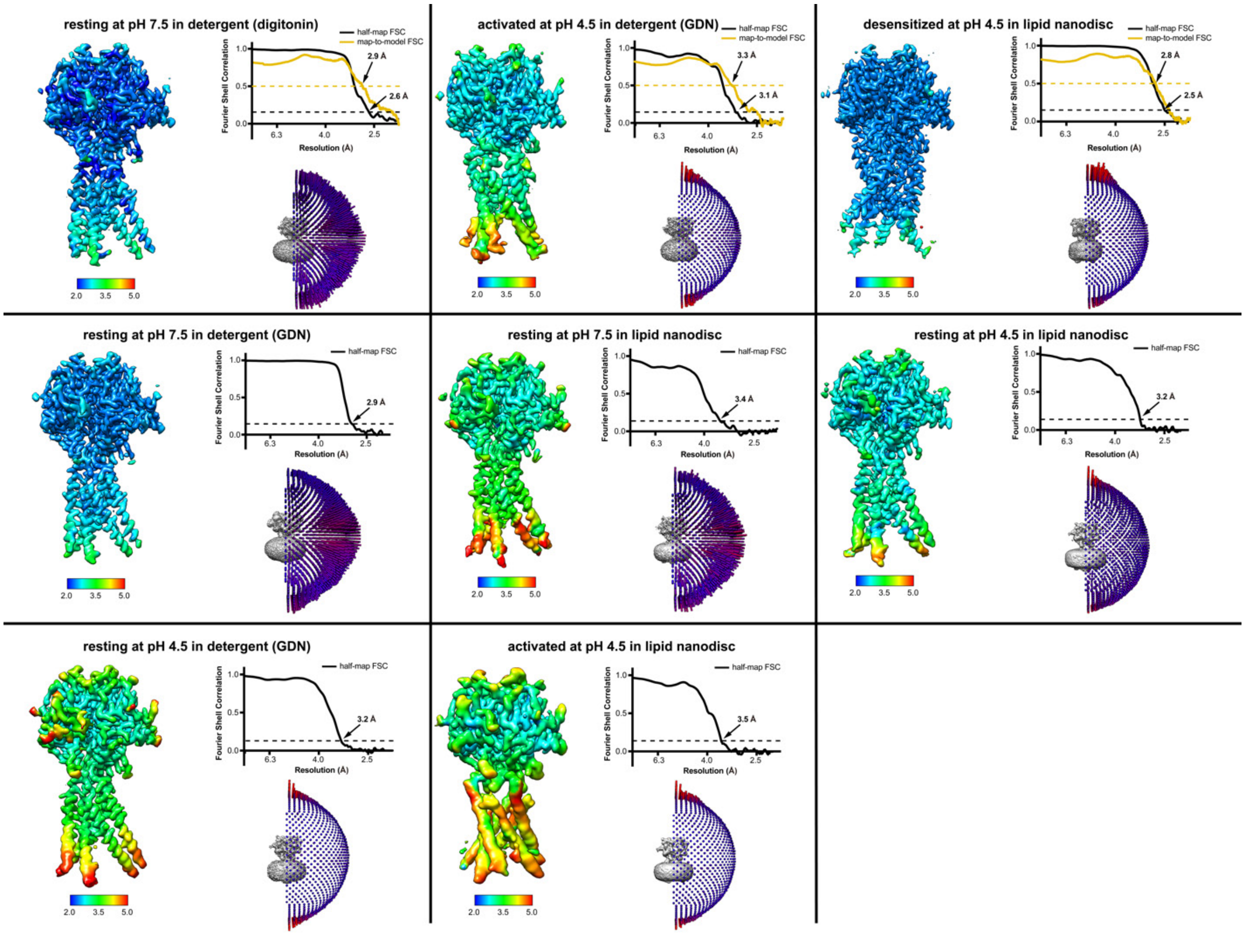

Fig. S8. Cryo-EM map analyses. Estimations of local resolution, half-map FSC curves, map-to-model FSC curves, and the angular distributions of particles used in the final reconstructions are shown. Details can be found in Methods. The best (final) reconstructions of the resting, activated and desensitized conformations are in the top panels.

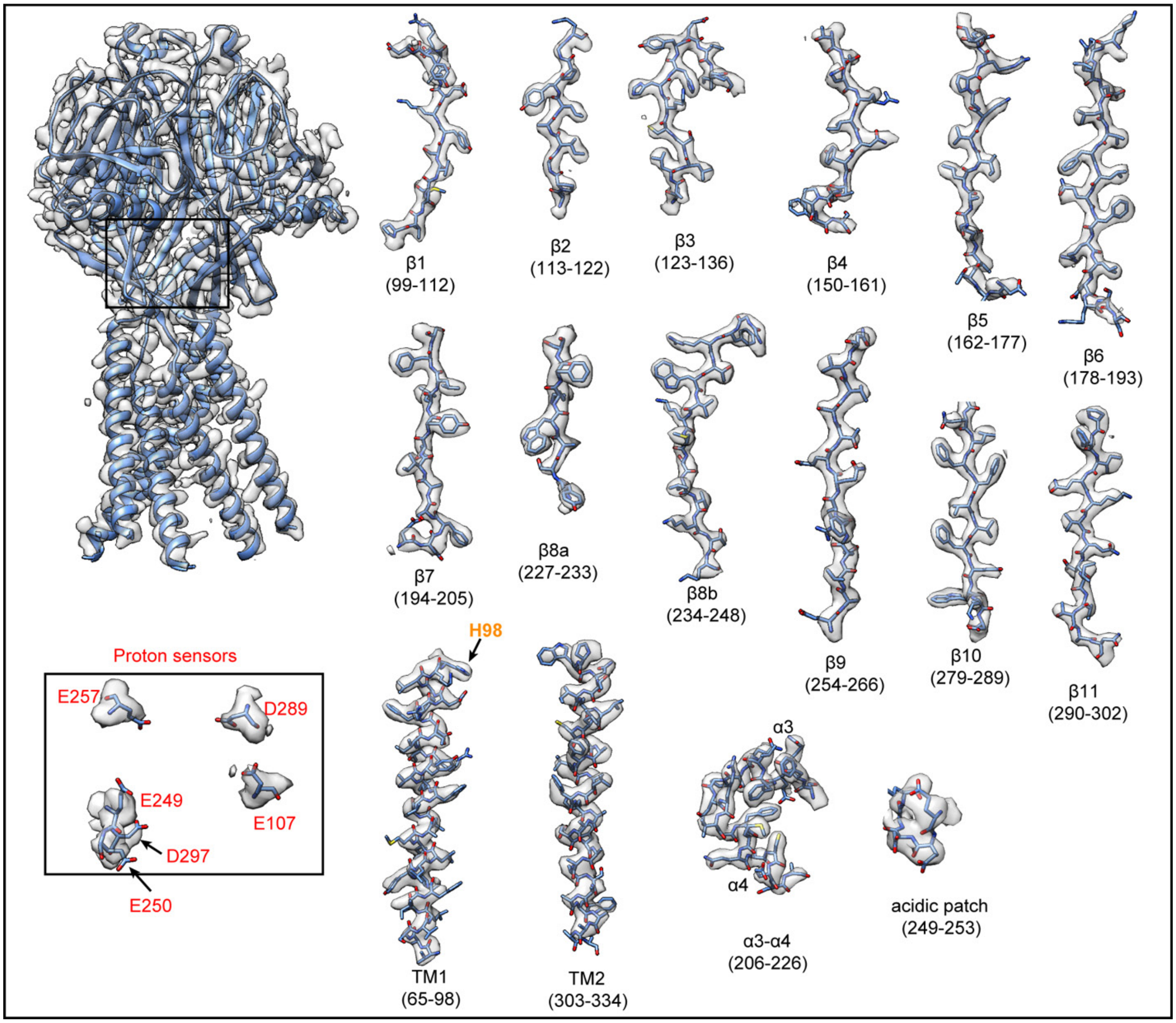

Fig. S9. Cryo-EM density of the resting conformation structure. Densities (semi-transparent surface rendering, 8 σ contour) for indicated regions are shown in the context of the atomic model (sticks).

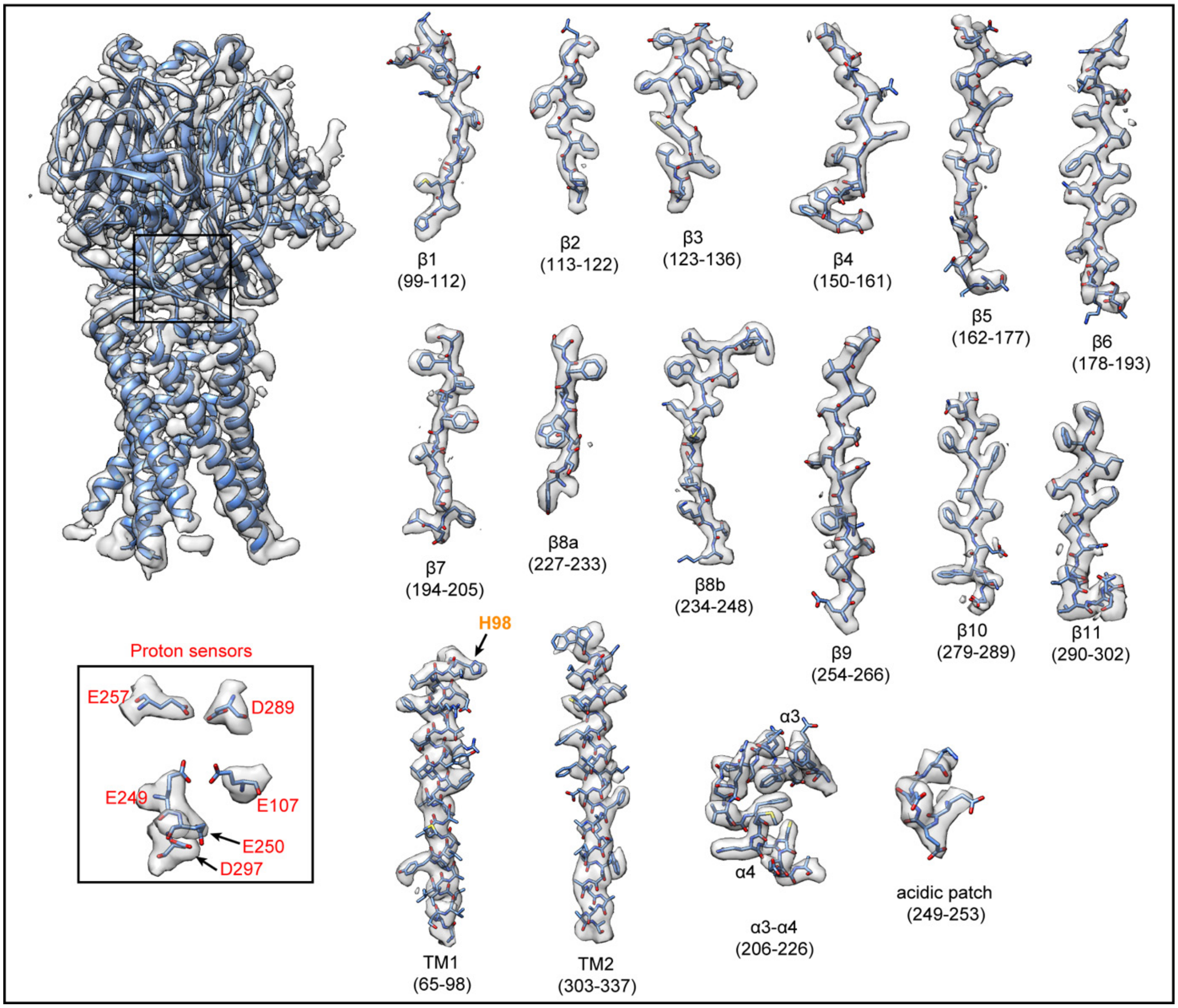

Fig. S10. Cryo-EM density of the activated conformation structure. Densities (semi-transparent surface rendering, 8 σ contour) for indicated regions are shown in the context of the atomic model (sticks).

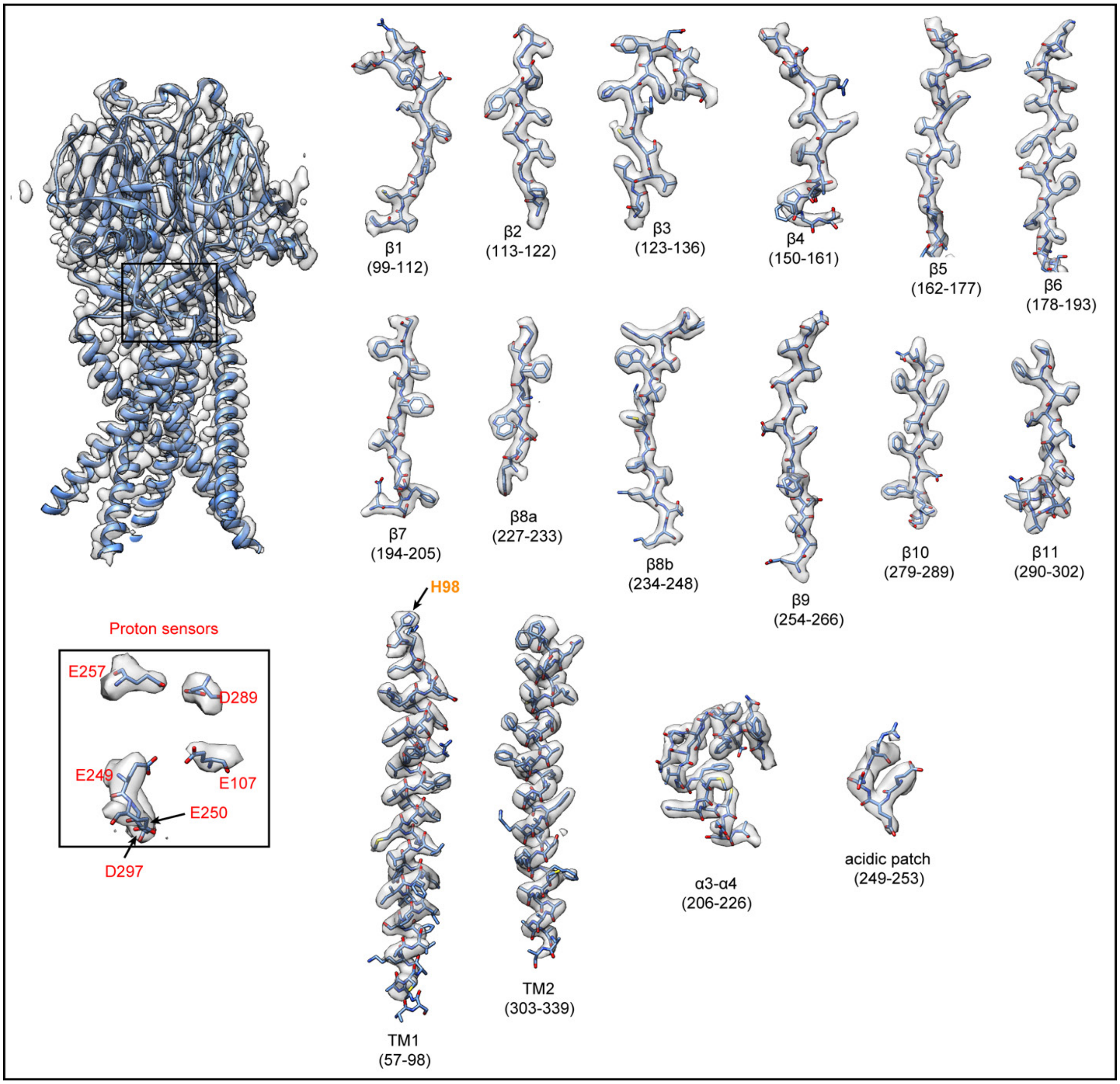

Fig. S11. Cryo-EM density of the desensitized conformation structure. Densities (semi-transparent surface rendering, 8 σ contour) for indicated regions are shown in the context of the atomic model (sticks).

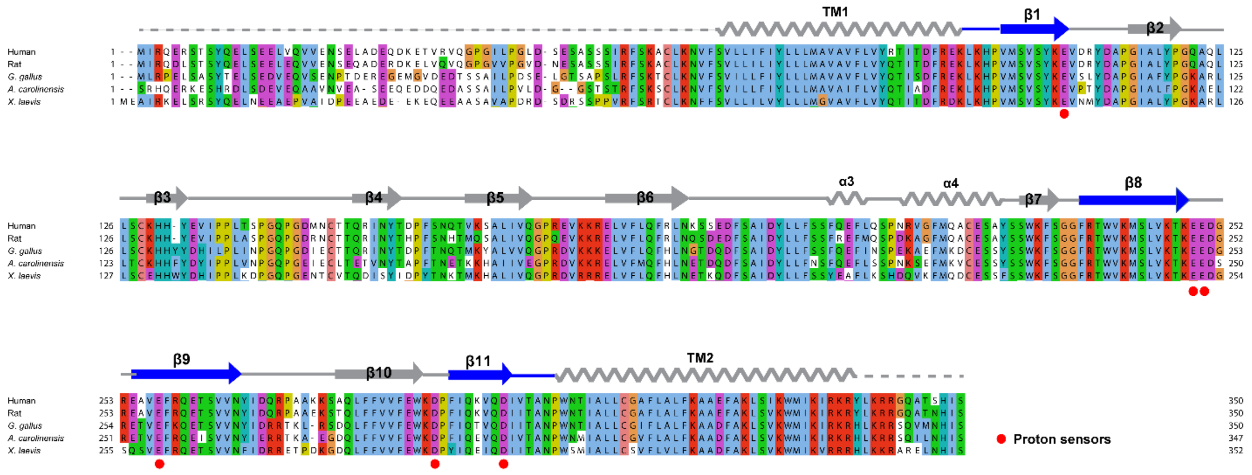

Fig. S12. Sequence comparison of ASOR proteins. The amino acid sequences of human, *R. norvegicus*, *G. gallus*, *A. carolinensis* and *X. laevis* ASOR (TMEM206) proteins are aligned and colored according to the ClustalW convention (UniProt accession numbers: Q9H813, Q66H28, E1C5B3, G1KFB8 and Q0IHD6, respectively). Secondary structures are indicated with cylinders representing α-helices, arrows representing beta sheets, solid gray lines representing structured loops, and dashed gray lines representing disordered regions. Acidic amino acids identified to have a role in proton sensing are indicated. Strands of the β-sheet-coupling domain are colored blue.

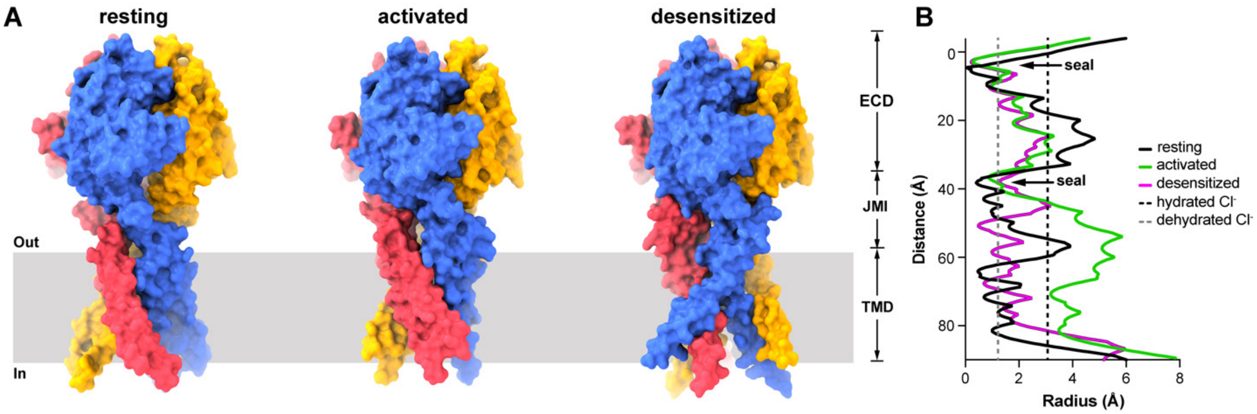

Fig. S13. Contraction of the ECD at acidic pH. (**A**) The molecular surfaces of the resting, activated, and desensitized conformations are shown, with individual subunits colored separately. (**B**) Radial dimensions along the symmetry axis of the channel in the three conformations, calculated as the minimal distance to the nearest van der Waals protein contact, as in Figure 2D, but showing dimensions throughout the ECD. Seals in each of the structures prevent ions from passing through the ECD (arrows).

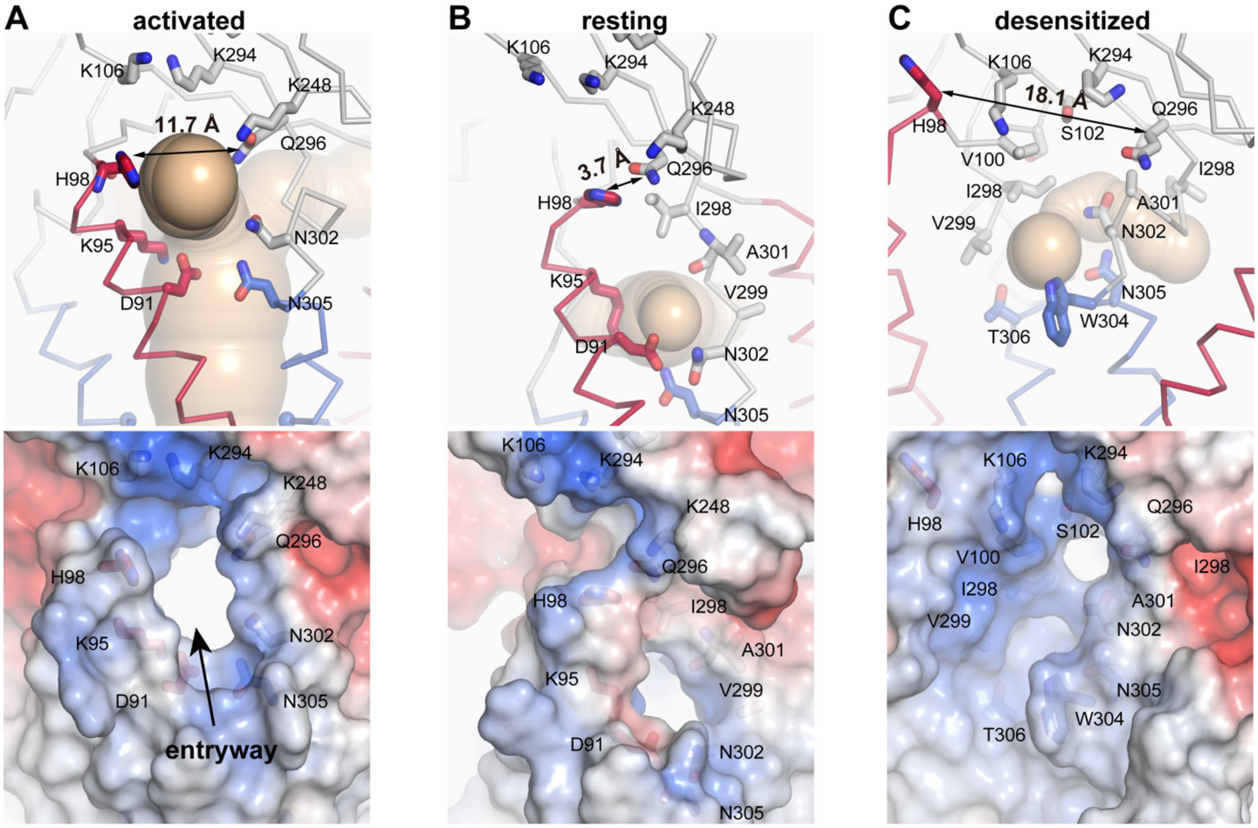

Fig. S14. Fenestrations in the activated, resting and desensitized conformations. (**A–C**) In the upper panels a solvent accessible surface (fenestration) leading to the symmetry axis of the pore is depicted (wheat color) for each structure. Surrounding residues are shown as sticks. Only the fenestration in the activated conformation (the entryway) is connected to an aqueous pore in the TMD through which ions could permeate. Distances between His98 (H98) and Gln296 (Q296) are denoted to indicate the dramatic changes in the vicinity of the entryway. The lower panels show the electrostatic surfaces of the same regions as the upper panels (red, -5 kT e^{-1}; blue, $+5$ kT e^{-1}). (A) is from Figure 4 and is shown for comparison with resting (B) and desensitized structures (C) from the same perspective.

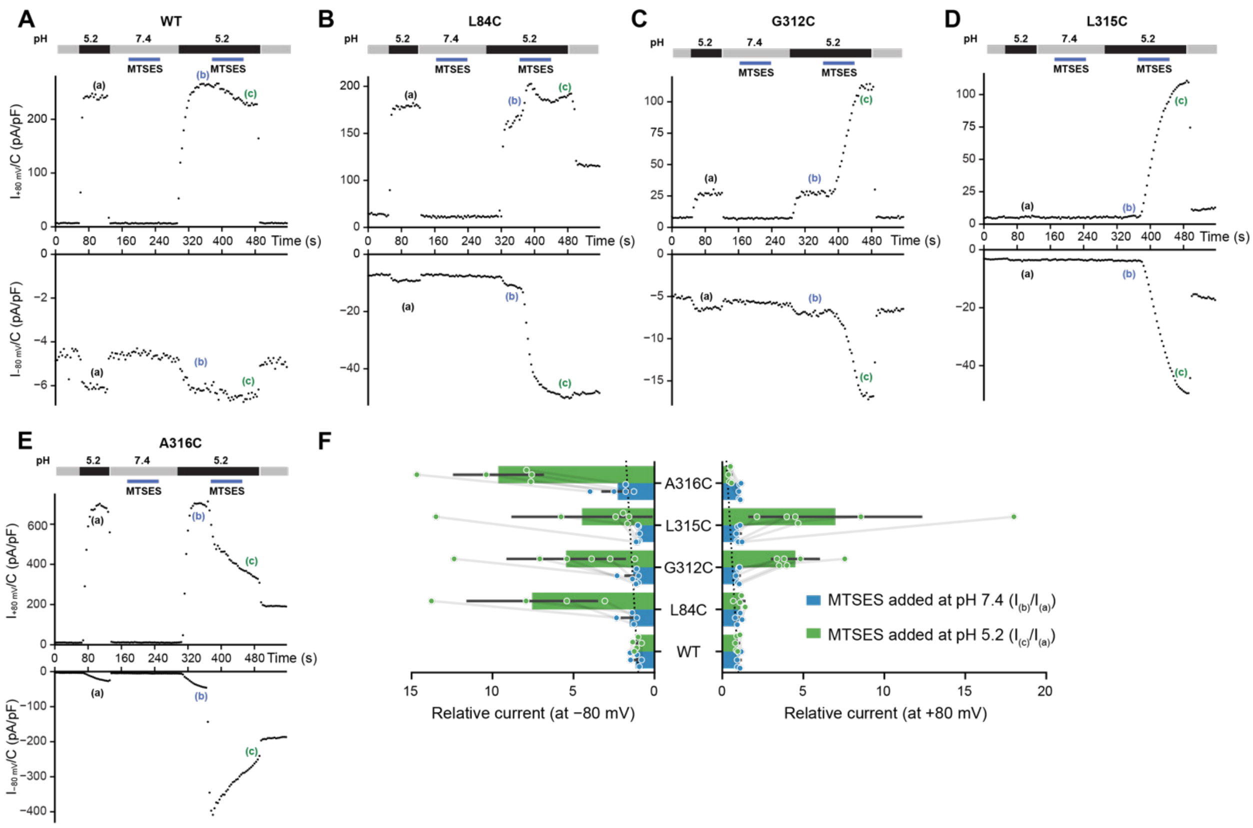

Fig. S15. Cysteine accessibility measurements. (**A–E**) Representative time courses obtained from individual cells expressing wild type (WT) or mutant ASOR channels. Voltage ramps from −80 mV to +80 mV were repeatedly applied every 4 s to cells in whole-cell configuration to measure ASOR current. After pipet break-in and measurement at pH 7.4, cells were exposed to a pH 5.2 bath solution to assess currents from channels prior to the addition of MTSES (timepoint 'a'). The external pH was then stepped back to pH 7.4 and 1 mM MTSES was applied for 80 s (blue bar). Unreacted MTSES was removed by perfusion and current was assessed again at pH 5.2 (timepoint 'b'). Then, 1 mM MTSES was applied at pH 5.2 for 80 s, unreacted MTSES was removed by perfusion, and the current was measured at pH 5.2 (timepoint 'c'). (**F**) Currents at pH 5.2 following the addition of MSTES at pH 7.4 (blue, at timepoint 'b') or pH 5.2 (green, at timepoint 'c') for the various mutants, shown for −80 mV and +80 mV and normalized to currents measured at pH 5.2 before MTSES addition (timepoint 'a'). Measurements from the same cell are connected with transparent gray lines.

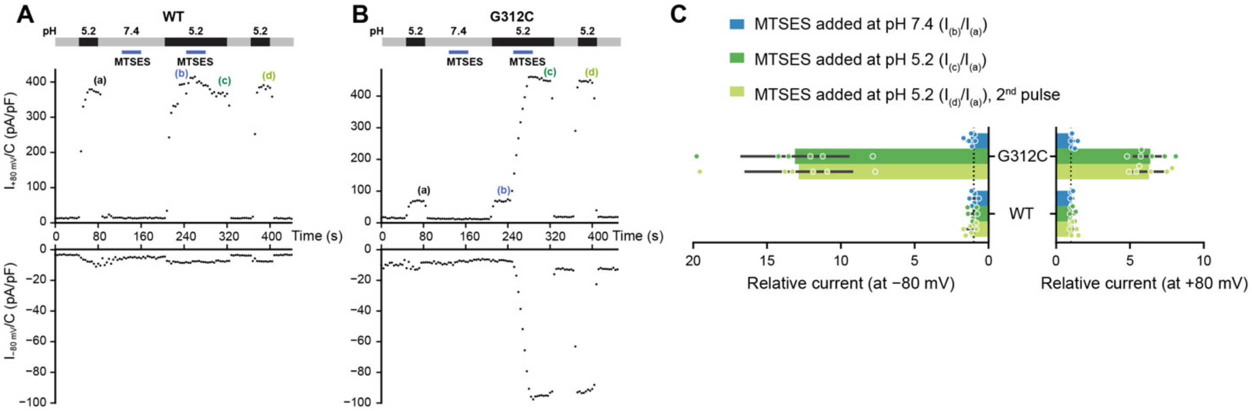

Fig. S16. Accessibility measurements of G312C demonstrate irreversible modification by MTSES. (**A–B**) Representative time courses obtained from individual cells expressing wild type (WT) or G312C ASOR channels. Measurements were obtained as in Fig. S15A–E, with an additional pulse of pH 5.2 (timepoint 'd') to demonstrate that the observed effects of MTSES treatment at pH 5.2 persist after extensive washout. (**C**) Current recorded at pH 5.2 following the addition of MSTES at pH 7.4 (blue, timepoint 'b'), pH 5.2 after removal of MTSES from the bath (green, timepoint 'c'), or after an additional pulse (light green, at timepoint 'd'). Data are shown for −80 mV and +80 mV and normalized to currents measured at pH 5.2 before MTSES addition (timepoint 'a').

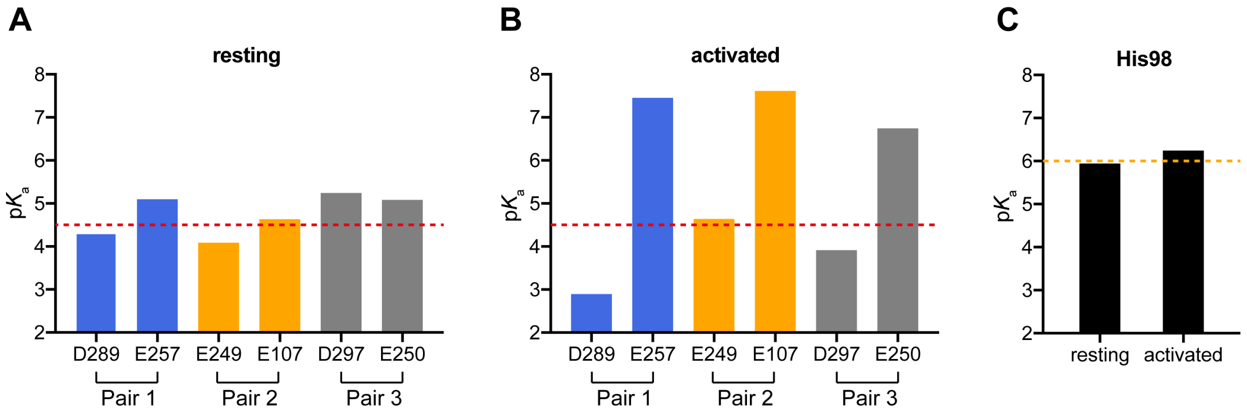

Fig. S17. Estimated pK_a values of acidic cluster residues and His98. (**A–B**) Estimated side chain pK_a values for acidic cluster residues in the resting and activated structures. Dashed lines represent standard side chain pK_a values for free amino acids. The shifted pK_a values in the activated structure suggest that one side chain per pair is protonated (which one may be arbitrary because the proton would be shared in the interaction). (**C**) Estimated side chain pK_a values for His98 in the resting and activated structures, which are only marginally shifted from the standard value.

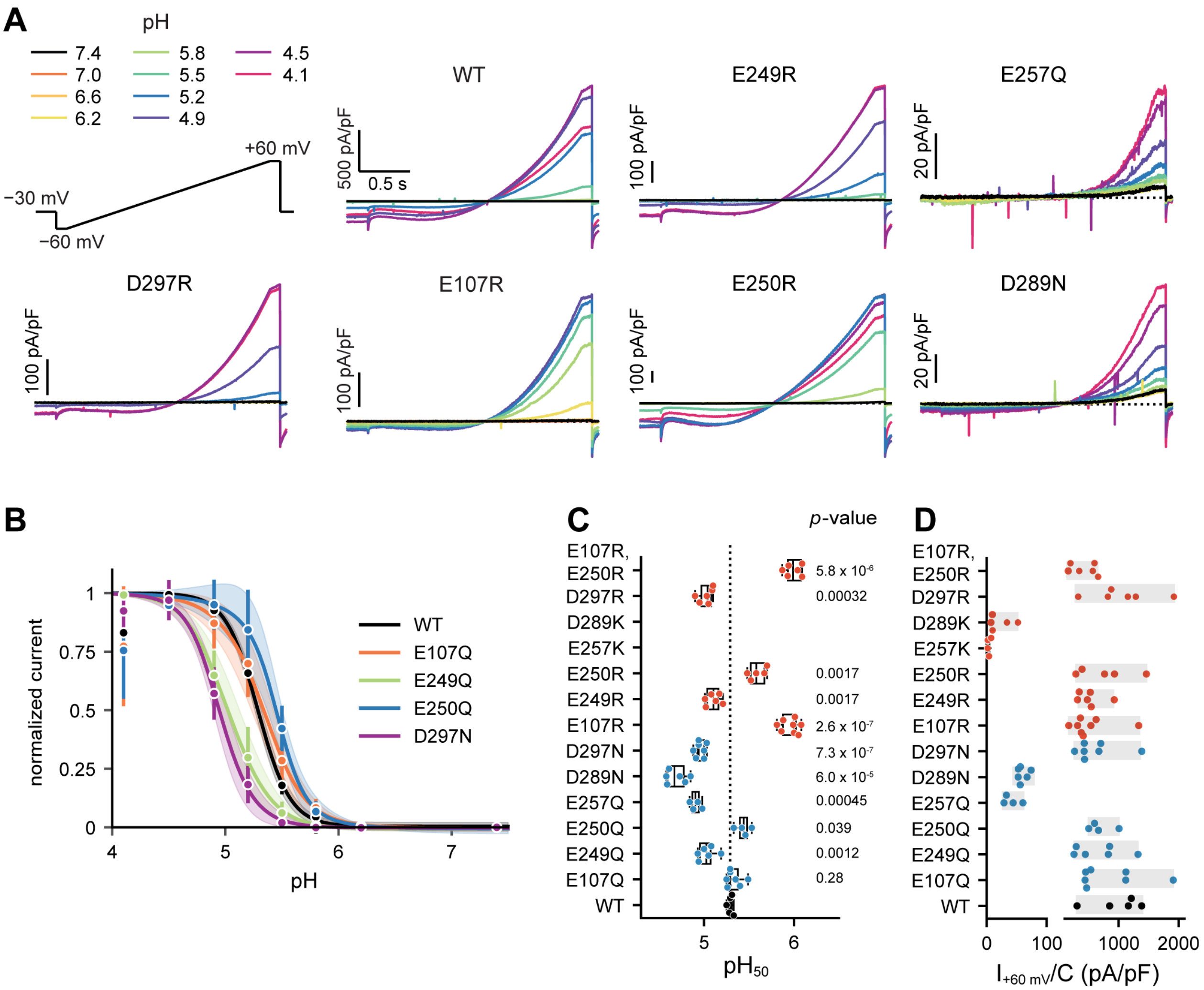

Fig. S18. Data for acidic cluster mutants. (**A**) Voltage ramp protocol (upper left) and representative current traces for data shown in Figure 4F-G. Currents were elicited from voltage-ramps applied to HEK293 cells expressing WT and mutant channels at the indicated extracellular pH values. Voltage ramps from −60 mV to +60 mV were applied every 5 s and bath pH was stepped from 7.4 to progressively more acidic values (50 s per step). (**B**) pH-dependence of current for additional mutations of the acidic cluster residues, calculated and depicted as in Figure 4F. (**C**) pH_{50} values for all the mutants tested, depicted as in Figure 4F. Charge neutralization mutations are shown in blue and charge-reversal mutations are red. Two-tailed Welch's t-Test *p*-values (false discovery rate controlled by Benjamini-Hochberg procedure) for mutants relative to the WT channel are shown. (**D**) Currents observed at +60 mV, normalized to cell capacitance, for the constructs in (C). Each data point corresponds to one transfected cell.

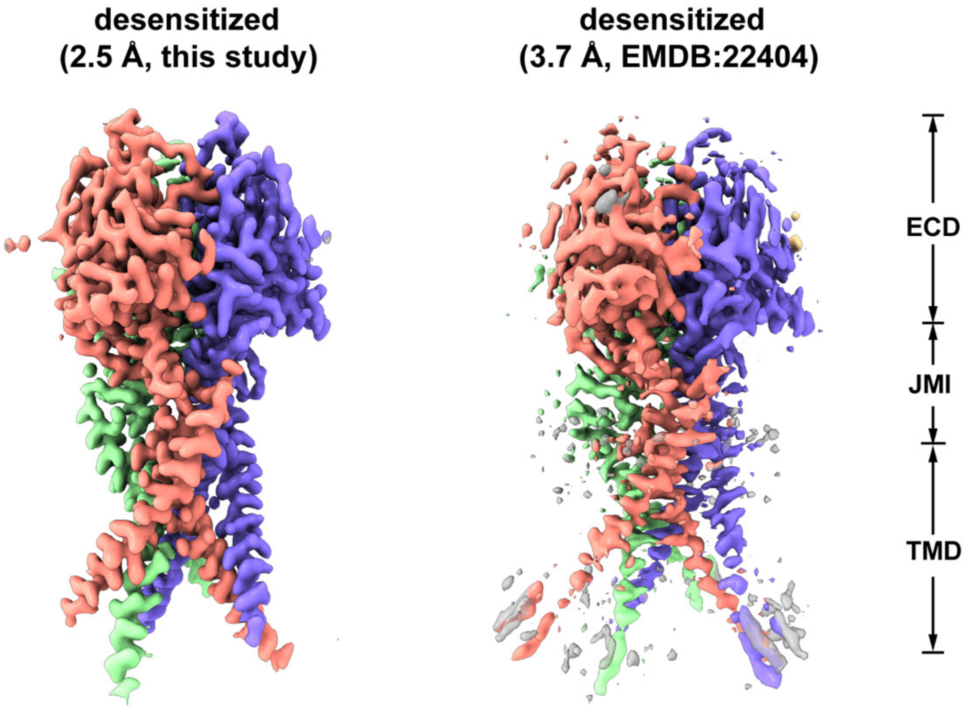

Fig. S19. Cryo-EM structures of desensitized conformations. Comparison of the desensitized map with the previously published map (EMDB: 22404, PDB: 7JNC) (*14*). The main chain RMSD of the corresponding atomic structures is 0.92 Å.

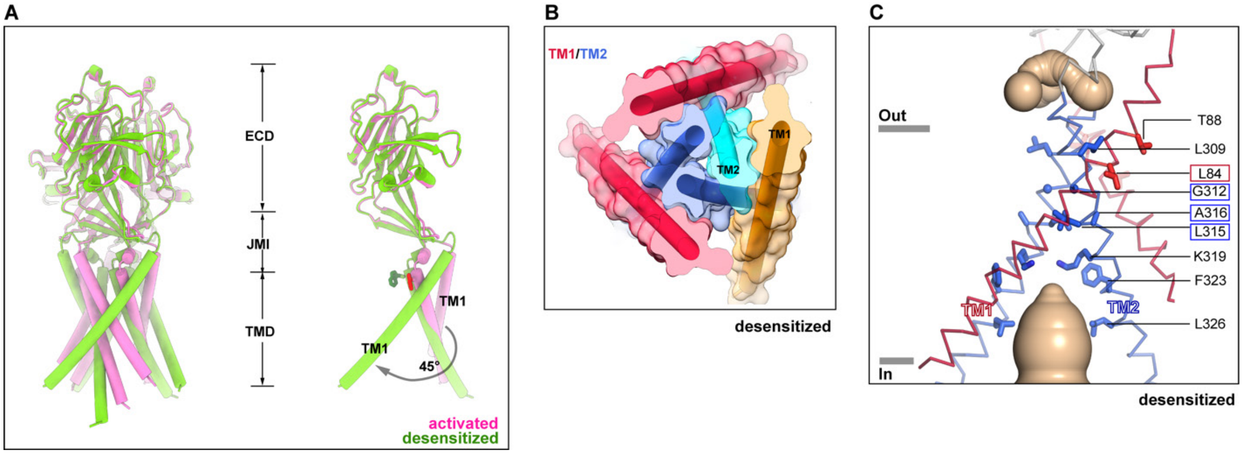

Fig. S20. The desensitized structure is a non-conductive conformation. (**A**) Comparison of the activated (pink) and desensitized (green) conformations. An overall comparison is shown in the left panel; the right panel compares the conformations of an individual subunit. The locations of Trp304 (right, sticks) are shown to indicate the vertical positions of TM2. (**B**) TMD of the desensitized conformation viewed from the extracellular side, with a semi-transparent molecular surface, depicted as in Figure 2A-B. TM1 of one subunit (e.g. orange) flanks TM2 of a neighboring subunit (e.g. dark blue) rather than its own TM2 helix (cyan) in the desensitized conformation. (**C**) The TMD of the desensitized structure from the side, depicted as in Figure 3A-B. Narrow dimensions along the axis of symmetry occlude solvent. Amino acids that form the walls of the activated pore are shown as sticks and labeled.

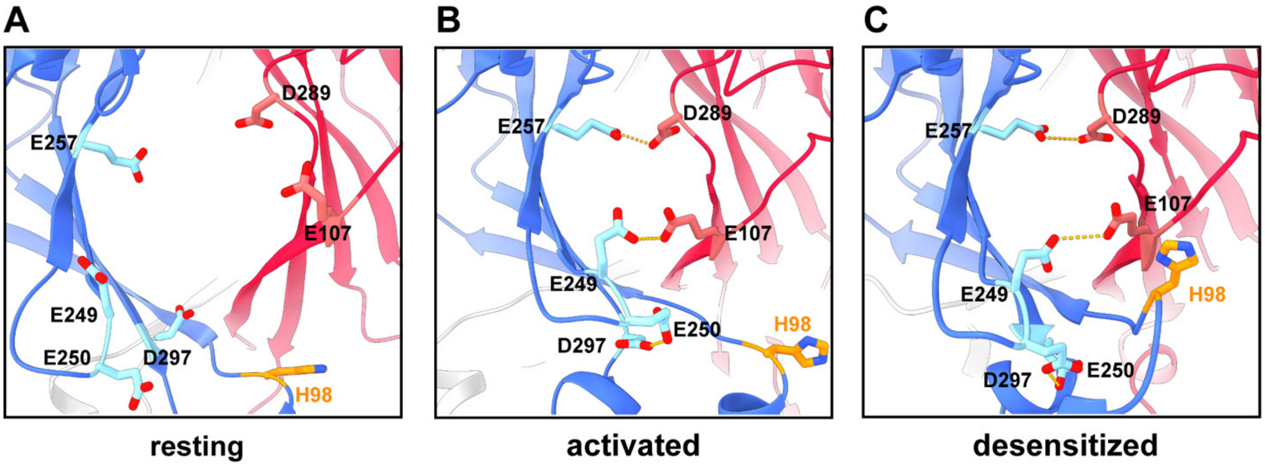

Fig. S21. The acidic cluster and His98 in the structures. (**A**-**C**) Close-up views of an acidic cluster and His98 in the resting, activated, and desensitized conformations. The acidic residues and His98 (orange) are shown as sticks. Carboxyl-carboxylate pairings in the activated and desensitized structures (B and C) are indicated by dotted lines. His98 is 14.0, 11.3, and 4.5 Å from the nearest acidic residue in structures, respectively (measured as the shortest distance bewteen side chain atoms), too far for direct interactions.

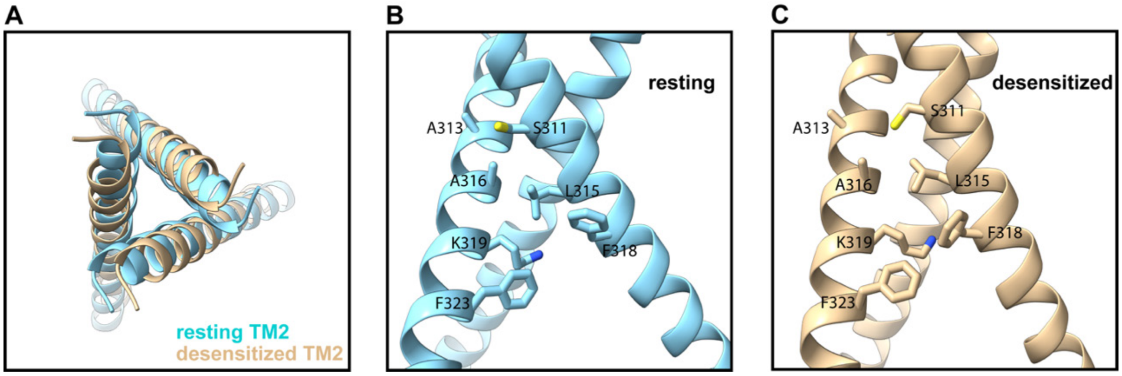

Fig. S22. The resting and desensitized conformations contain similar bundles of TM2 helixes. (**A**) Superposition of TM2 bundles in the resting (cyan) and desensitized (tan) conformations, viewed from extracellular side. (**B-C**) Close-up views of the TM2 bundles in resting (B) and desensitized (C) conformations, with interacting residues depicted as sticks; shown from the side.

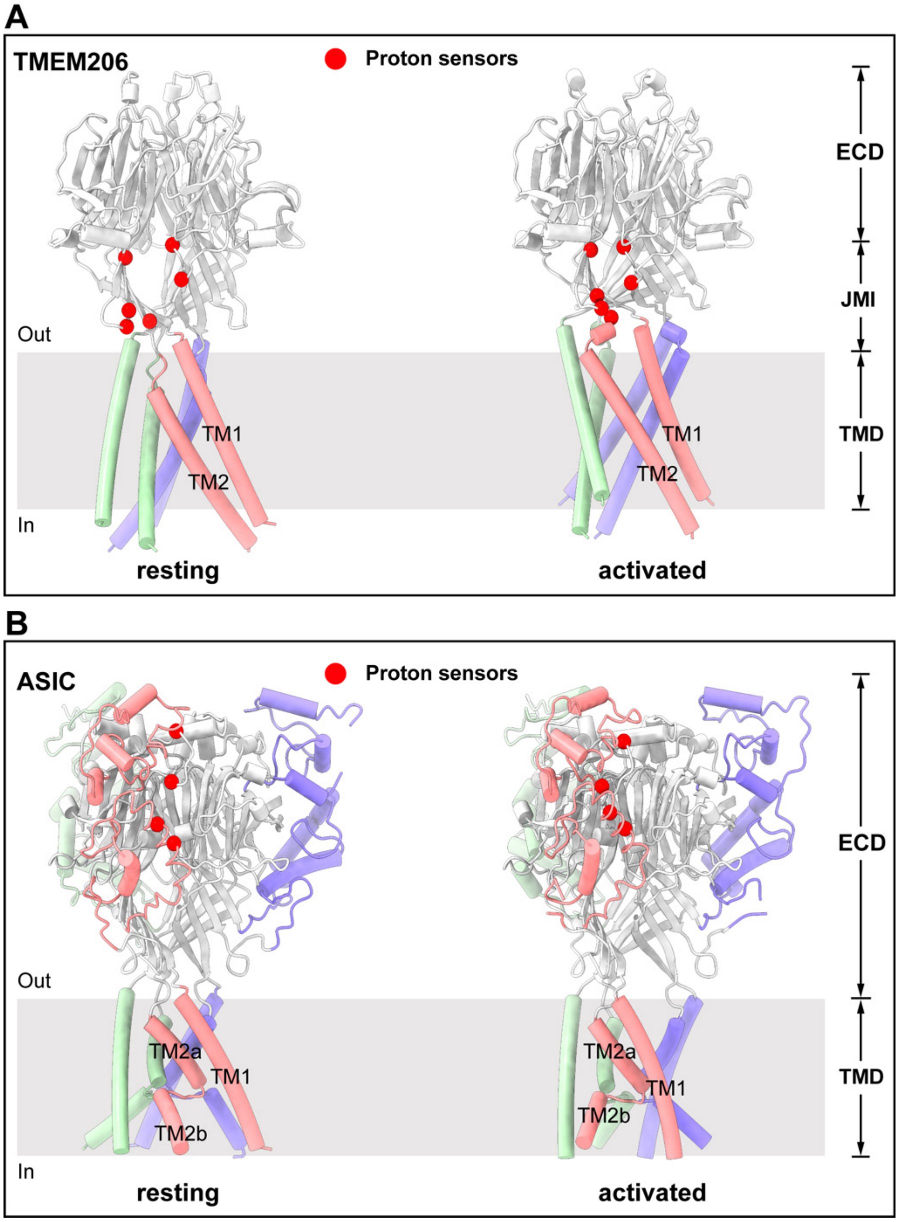

Fig. S23. Comparison between ASIC and ASOR. (**A**) Resting and activated conformations of ASOR viewed from the membrane. The TMD of each subunit is colored separately. Acidic cluster residues are indicated as red spheres. (**B**) Resting (PDB: 5WKU) and activated (PDB: 4NTW) conformations of the ASIC channel. α-helical regions in the ECD that do not have counterparts in ASOR are colored. Acidic amino acids that function as proton sensors are denoted by red spheres.

	Resting (pH 7.5, digitonin) PDB: xxxx EMD-xxxxx	Activated (pH 4.5, GDN) PDB: xxxx EMD-xxxxx	Desensitized (pH 4.5, nanodisc) PDB: xxxx EMD-xxxxx	Resting (pH 7.5, GDN) EMD-xxxxx	Resting (pH 7.5, nanodisc) EMD-xxxxx	Resting (pH 4.5, GDN) EMD-xxxxx	Resting (pH 4.5, nanodisc) EMD-xxxxx	Activated (pH 4.5, nanodisc) EMD-xxxxx
**Data collection and processing**								
Microscope	FEI Titan Krios (PNCC)	FEI Titan Krios (PNCC)	FEI Titan Krios (MSK)	FEI Titan Krios (MSK)	FEI Titan Krios (MSK)	FEI Titan Krios (PNCC)	FEI Titan Krios (MSK)	FEI Titan Krios (MSK)
Camera	Gatan K3	Gatan K3	Gatan K3	Gatan K3	Gatan K3	Gatan K3	Gatan K3	Gatan K3
Magnification	29,000×	22,500×	22,500×	22,500×	29,000×	22,500×	22,500×	22,500×
Voltage (kV)	300	300	300	300	300	300	300	300
Electron exposure (e^{-}/Å^2)	48	37	71	36	55	37	71	71
Defocus range (μm)	−1.0 ~ −2.3	−1.0 ~ −2.3	−1.0 ~ −2.6	−1.0 ~ −2.6	−1.0 ~ −2.6	−1.0 ~ −2.3	−1.0 ~ −2.6	−1.0 ~ −2.6
Pixel size (Å)	0.826 (0.413)*	1.059 (0.530)*	1.064 (0.532)*	1.064 (0.532)*	0.826 (0.413)*	1.059 (0.530)*	1.064 (0.532)*	1.064 (0.532)*
Software	RELION 3.1, cryoSPARC v2	RELION 3.1, cryoSPARC v2	RELION 3.0, cryoSPARC v2	RELION 3.1, cryoSPARC v2	RELION 3.0, cryoSPARC v2	RELION 3.0, cryoSPARC v2	RELION 3.0, cryoSPARC v2	RELION 3.0, cryoSPARC v2
Symmetry imposed	C3	C3	C3	C3	C3	C3	C3	C3
Initial particle images (no.)	612,580	1,044,859	3,097,510	975,292	115,204	1,044,859	3,097,510	3,097,510
Final particle images (no.)	60,374	36,772	157,906	391,364	26,171	36,051	109,714	43,714
Overall map resolution (Å)	2.6	3.1	2.5	2.9	3.4	3.2	3.2	3.5
FSC threshold 0.143								
Local map resolution range (Å)	2.0-3.5	2.5-4.5	2.0-3.5	2.2-3.5	3.0-5.0	2.5-4.5	2.5-5.0	3.0-5.0
**Refinement**								
Software	Phenix 1.13 real-space-refine	Phenix 1.13 real-space-refine	Phenix 1.13 real-space-refine					
Initial model (PDB code)	N/A	N/A	N/A					
Model resolution (Å)	2.9	3.3	2.8					
FSC threshold 0.5								
Map sharpening *B* factor (Å^2)	−30	-30	-30					
Model composition								
Non-hydrogen atoms	6,630	6,642	6,954					
Protein residues (#)	810 (65-334)	819 (65-337)	849 (57-339)					
Ligands	12 (NAG)	12 (NAG)	12 (NAG)					
Water	0	0	21					
B factors (Å^2)								
Protein	87.1	74.8	71.6					
Ligand/Water	111.9	79.8	94.6/63.1					
R.m.s. deviations								
Bond lengths (Å)	0.004	0.004	0.005					
Bond angles (°)	0.565	0.564	0.710					
Validation								
MolProbity score	1.30	1.45	1.14					
Clashscore	3.35	5.34	3.19					
Poor rotamers (%)	0.00	0.00	0.00					
Ramachandran plot								
Favored (%)	97.01	97.05	97.63					
Allowed (%)	2.99	2.95	2.37					
Disallowed (%)	0.00	0.00	0.00					

Table S1. Data collection, refinement, and validation statistics. *Super-resolution pixel size; #Starting and ending residues.

Movie S1. TMD metamorphosis upon activation. This movie shows a morph between the resting and activated structures. The view is of the TMD, from the extracellular side. TM1 helices are light brown and TM2 helices are green. α-helices are shown as ribbons with amino acids as sticks.

Movie S2. Channel activation. This movie shows a morph between the resting and activated structures. The channel is depicted in cartoon representation, from the side. TM1 helices are light brown and TM2 helices are green, with amino acids on them drawn as sticks. The β-sheet-coupling domain is blue and residues of the acidic cluster closest to the viewer are drawn as magenta sticks.
